# A Short Review on Polymeric Biomaterials as Additives for Lubricants

**DOI:** 10.3390/polym13081333

**Published:** 2021-04-19

**Authors:** Gobinda Karmakar, Koushik Dey, Pranab Ghosh, Brajendra K. Sharma, Sevim Z. Erhan

**Affiliations:** 1 Natural Product and Polymer Chemistry Laboratory, Department of Chemistry, University of North Bengal, Darjeeling 734013, India; gobinda.chem@gmail.com (G.K.); koushikdey00@gmail.com (K.D.); pizy12@yahoo.com (P.G.); 2 Illinois Sustainable Technology Center, Prairie Research Institute, University of Illinois at Urbana-Champaign, 1 Hazelwood Drive, Champaign, IL 61820, USA; 3 Agricultural Research Service Eastern Regional Research Center, United States Department of Agriculture, Wyndmoor, PA 19038, USA

**Keywords:** vegetable oils, polymerization, viscosity index, pour point, tribological performance

## Abstract

With increasing environmental concerns and the depletion of petroleum resources, the development of lubricant additives from bioresources has attracted much attention recently. In this review, we reported a few polymers and polymer composites that are synthesized from vegetable oils (soybean oil, sunflower oil, rice bran oil, and castor oil) and used as multifunctional additives in the formulation of eco-friendly lubricant compositions. We mentioned the preparation of vegetable oil-based homo- and copolymers and their characterization by different spectral techniques (FTIR/NMR). The average molecular weights of the polymers are determined by gel permeation chromatography (GPC). Performance evaluations of the polymeric materials mainly as a viscosity index improver (VII), pour point depressant (PPD), and most importantly antifriction additives when blended with lubricating base oils are indicated. Standard ASTM methods have been applied to evaluate their performances. The findings have shown that all the additives discussed are non-toxic, biodegradable, and showed excellent performances compared to commercial petroleum-based additives.

## 1. Introduction

The synthesis of different polymeric materials from bioresources and their application in diverse fields is a timely area of research because of several environmental impacts of commercially available nonbiodegradable polymers. Vegetable oil (VO), due to its biodegradability, low ecotoxicity, and renewability, can be used as an excellent starting material to prepare different polymeric compounds as an alternative to synthetic petroleum-based polymers in different sectors [1,2,3,4]. The application of biopolymers has been explored in different industrial sectors and products such as paints, coatings, ink resins, lubricants, and others. Good lubrication in internal combustion engines is essential to prevent energy loss, lengthen the lifetime of engines, and reduce environmental pollution. Lubricating oils are unable to meet all the requirements of modern engines, so it is essential to optimize the performance of lubricants by combining them with suitable additive packages. Additives, needed only in tiny amounts, have a very important role in improving some basic performances of lubricants such as antiwear, pour point, viscosity index, thermo-oxidative stability, etc. [5,6,7]. Commercially available synthetic additives prepared from fossil fuel resources (e.g., acrylate-based polymers, polycaprolactones [PCL], polyesteramides [PEA], aliphatic copolyester, ZDDP), although cheaper than biopolymers, are extremely harmful to the biosphere. They are ecotoxic and not readily biodegradable. The environmental hazards of the ZDDP additive have been well-studied in vertebrate and invertebrate species. They are considered to be toxic to aquatic wildlife with long-lasting effects. ZDDP contains sulfur, phosphorus, and zinc, which produce harmful emissions from engines during the tribo-chemical process [8]. An additive like ZDDP does not work well under extreme conditions of pressure and temperature. Bioadditives prepared from biooils/bioresources have the advantage over petroleum-based additives because of their eco-friendly character, multifunctional behavior, and superior tribological properties [9,10].

**Figure 1:**
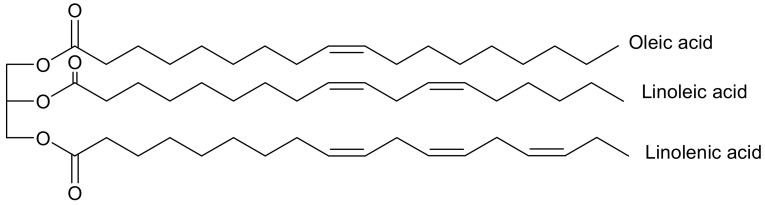
General structure of triglyceride esters of edible vegetable oils.

Vegetable oil triglycerides contain long-chain fatty acids (14–22 carbons chain) with varying levels of unsaturation (Figure 1). Most vegetable oils have separate regions of nonpolar and polar groups in the same molecule. Due to the presence of polar groups, vegetable oils are amphiphilic and therefore can be used as an excellent resource to prepare both boundary and hydrodynamic lubricants [11,12]. They have many promising natural properties including good lubricity, good resistance to shear, a high flash point, a high viscosity index, and a low evaporative loss over mineral oils [13,14,15]. Therefore, the application of vegetable oils as feedstock to prepare bioadditives is compelling and has come into focus recently. However, because of a few shortcomings such as a high price, poor fluidity at cold temperatures, and low thermal and oxidation stability, mainly due to structural “double bond” elements in the fatty acid part and the “β-CH group” of the alcoholic components, raw vegetable oils cannot be used as a base stock/additive in lubricants [16]. This problem can be mitigated by chemically modifying vegetable oil before its application as lubricants (base stocks/additives). Among the several methods of chemical derivations [17,18,19,20], polymerization is an easy and economic synthetic technique that removes the olefinic bonds of VO/fatty esters and mitigates the shortcomings of their application by improving thermo-oxidative stability [20,21].

The vegetable oils/derivatives having a higher percentage of unsaturation are subjected to polymerization in different modes such as free radical, cationic, olefin metathesis, condensation polymerization, and recently, atom transfers radical polymerization (ATRP) to prepare materials with excellent additive performances [20,22]. The morphology and therefore performance of the additive are also dependent on the polymerization method used. Free radical polymerization is very effective in preparing polymeric additives that have better performances and therefore is mostly used to prepare lubricant additives [22,23]. In the polymerization, VO, VO-derived fatty acids/esters, or VO mixed with other materials can be used as monomers [20,22,23,24].

Most of the research is directed towards the synthesis of biolubricants by blending nonbiodegradable additives with chemically modified vegetable oils [25,26]. Yet, the formulation will be expensive since modified vegetable oils are costly compared to petroleum base stocks, and their abundance is insufficient to meet the present demand for lubricants worldwide. The blending of bioadditives from vegetable oils with mineral base stocks is currently a cost-effective and environmentally benign formulation of green lubricants. The lubricants additized with polymeric biomaterials showed an excellent tribological performance and improved the viscosity index properties and biodegradability [22,23,24,27,28,29]. The homopolymerization of functionalized triglyceride esters, free fatty acid esters of vegetable oils, or their copolymerization with suitable monomers produces biopolymers with improved additive performances and thermophysical and thermomechanical properties that have a wider range of applicability [29,30,31].

Our earlier publications mentioned the application of soybean oil, sunflower oil, castor oil, rice bran oil, palm oil, and olive oil-based polymers as multifunctional and thermally stable additives for mineral oil-based lubricants [24,27,28,29,32,33,34]. Biresaw et al. [35] discussed the application of biobased polyesters as an extreme pressure additive in mineral (150 N) and refined soybean base oils. The use of poly (hydroxy thioether) vegetable oil derivatives as antiwear/antifriction additives for eco-friendly lubricants has been described by Erhan et al. in U.S. Patent 7,279,448 B2 (2007) [36]. Landis [37] reported on the synthesis and evaluation of telomerized vegetable oil, sulfurized and phosphorus derivatives of telomerized vegetable oils, and combinations thereof for use as thermal-oxidative stability enhancers and viscosity improvers. Telomerization induces vegetable oil that contains no more than 4% polyunsaturated fatty acids to help enhance its thermo-oxidative stability. Recently, Nasser et al. [38] reported about the performance of the homopolymer of jojoba oil and its copolymers with different alkyl acrylates and α-olefins as additives in lubricating oil and found amazing results.

Polymer nanocomposites have excellent wear resistance characteristics when coated on machinery parts/other materials. There are several examples of tribological performances of polymer composites. The composites of ultrahigh molecular weight polyethylene (UHMWPE) have been used as coatings to modify the surfaces of different components to protect them from wear and corrosion [39]. The composite of UHMWPE reinforced with carbon nanotube (CNT) was found as effective boundary lubricants and reduce or eliminate the usage of harmful additives in the lubricating oils. These coatings have found their way into applications ranging from microelectromechanical systems (MEMS) to demanding tribological applications such as bearings and biomedical applications [40]. The polymer composites based on vegetable oils prepared by incorporating different organic or inorganic filler particles (e.g., silica, carbon nanotubes, clay platelets, etc.) are also widely applied in different sectors such as the automotive industry [41,42], medicine [43], coating technology [44], food industry [45], preparation of printing inks [46], agrochemicals [47], aerospace [48], and more. These are used as additives to improve the tribological performances of lubricants.

**Scheme 1:**
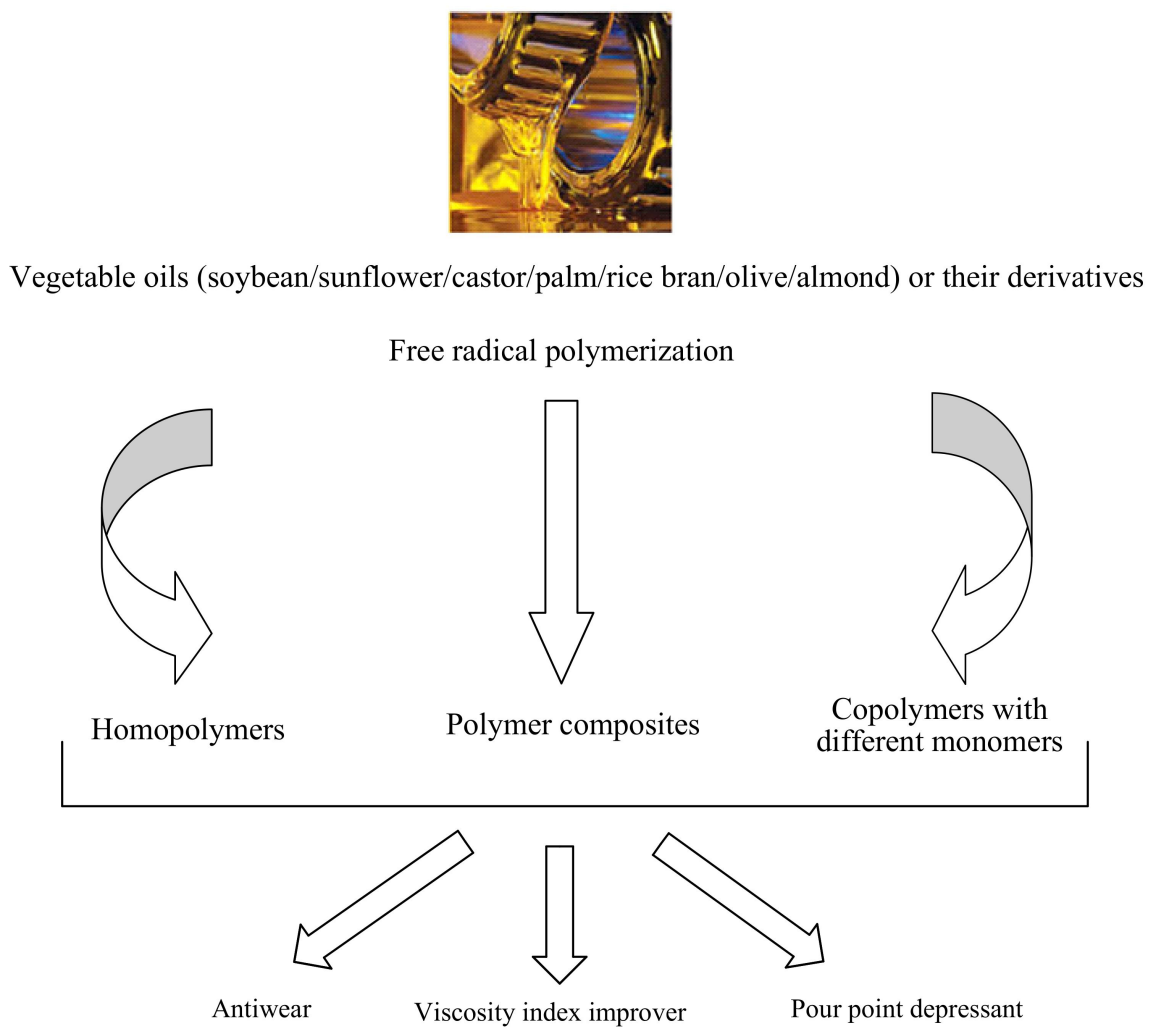
Pictorial representation of the overall work.

In this review article (Scheme 1), we discussed in detail different vegetable oil-based polymers/polymer composites that are used as multifunctional additives for environmentally benign lubricant formulation.

## 2. Vegetable Oils and Their Physicochemical Properties

The major constituent of vegetable oils are triglyceride esters (92–98%) of different long-chain fatty acids (saturated and unsaturated, Figure 1). The other constituents are polar lipids (phospholipids and galactolipids), monoacylglycerols, diacylglycerols, and minor amounts of free fatty acids and polyisoprenoids. Therefore, the physicochemical characteristics of vegetable oils largely depend on the fatty acid compositions in the glyceride moiety. The higher the percentage of unsaturation in the fatty acid chains, the more will be the scope of the functionalization/modification of VOs to improve their oxidative stability and cold flow properties. Therefore, VOs containing a higher percentage of unsaturation, such as soybean oil, sunflower oil, palm oil, castor oil, linseed oil, and rice bran oil, are important for preparing polymeric additives for lubricants. Among them, the nonedible vegetable oils such as neem, castor, mahua, rice bran, karanja, jatropha, and linseed, being comparatively less expensive, have an advantage over edible oils in producing additives for biofuel/biolubricants [27]. Table 1 gives a comparative look at the different fatty acid compositions with total percentages of unsaturation in different VOs.

The quality and performance of an additive depend largely on the physical properties of the VO from which it was derived. A vegetable oil having a higher viscosity index, flash point, thermo-oxidation stability, shear stability, and lower pour point and cloud point produce additives with enhanced performances [27]. The physical characteristics of different VOs are listed in Table 2.

## 3. Polymerization of Different Vegetable Oils/Derivatives, Their Characterization, and Application as Lubricant Additives

Polymerization is a unique technique through which the reactive olefinic bonds of VO/derivatives are involved, and therefore the oxidative stability and cold flow properties of the oils are improved. The homopolymerization of VO/derivatives or their copolymerization with suitable monomers can be performed in different ways, as mentioned above. Here, we have discussed the polymers/composites synthesized via the free radical polymerization pathway. Homopolymers of vegetable oil and their copolymers with different functional molecules (alkyl acrylates, long-chain alkenes, styrene) were prepared by thermal/microwave methods using radical initiators like azobisisobutyronitrile (AIBN), benzoyl peroxide (BZP) [24,28]. The characterizations were performed by different spectral (FTIR/NMR) techniques. The average molecular weights were determined by GPC. The characterization data (FTIR, NMR, GPC) of some polymers are mentioned in Table 3. The individual descriptions of the different vegetable oil-based polymers as lubricant additives are depicted below.

The IR and ^1^HNMR absorption peaks in the range 1732–1745 cm^−1^ and 4.031–4.9 ppm, respectively in Table 3 indicated the presence of ester carbonyl groups of the polymers. The C-O stretching vibrations of ester carbonyl groups of all the polymers appeared within the range 1051–1270 cm^−1^. The presence of the methylene and methyl groups of the fatty acid chains is also proved by the respective IR and ^1^HNMR absorption peaks in Table 3. The GPC data in the above table gives some idea about the average molecular weights of the biobased polymers. The values of the average molecular weight depend on the degree of polymerization, cross-linking of the monomers/unsaturated fatty esters, and the average size of polymer molecules. These further depend upon several factors such as the degree of unsaturation of different vegetable oils, the type of monomers (in case of copolymerization), and the methods and conditions applied on polymerization (time, ratio of monomers, initiators, temperature, etc.). The homopolymer of vegetable oils like SBO, SFO containing a higher percentage of unsaturation showed comparatively higher average molecular weights. When acrylate chains are introduced in the backbone of triglyceride esters of vegetable oils, the average molecular weights are increased due to a higher degree of polymerization which is reflected on their VI values, also mentioned later. Terpolymers have a comparatively low value of the average molecular weight, probably due to a shorter chain length of each polymer unit.

### 3.1. Polymers of Soybean Oil/Derivatives

After palm oil, soybean oil (SBO) is the second most abundant vegetable oil with a production of around 50 million metric tons of the 209.14 million metric tons of all vegetable oils produced in 2020–2021, according to statistical reports. The United States occupied the second position in the production of soybean oil after China. The unsaturation in SBO is around 85%, which makes it suitable for producing different polymeric additives. In our previous work, the homopolymer of soybean oil and its copolymers with different monomers, such as methyl acrylate (MA), 1-decene, and styrene, at different percentage levels have been synthesized by a thermal method using azobisisobutyronitrile (AIBN) as a radical initiator [24]. All the polymers showed excellent multifunctional performances as additives in lubricants. The viscosity index (VI), antiwear, and pour point (PP) properties of the base fluids were enhanced significantly by the addition of these additives. Polymerization increased the thermo-oxidative stability of soybean oil also. Table 4 shows the VI and PP values of lubricant compositions blended with additives at different percentages.

The data indicated that the VI values of the base oils blended with additives are higher compared to those without additives. The results also showed that the VI values gradually increase with an increase in the additive concentration of the base fluids. The homopolymer of SBO showed excellent results. The incorporation of styrene and 1-decene to SBO enhanced the VI values, whereas copolymers of SBO with MA (S-2 and S-3) showed the least VI value. Moreover, it was found that all the polymers have much higher VI values compared to commercially available acrylate-based or olefin polymers. The pour point value of the copolymer of soybean oil with MA exhibited a better pour point depressant (PPD) performance compared to the others. The soy-acrylate copolymers, due to their more polar nature, fight better against the formation of wax crystals and therefore show higher pour point values that are comparable with those of commercially available polymethacrylates.

**Figure 2:**
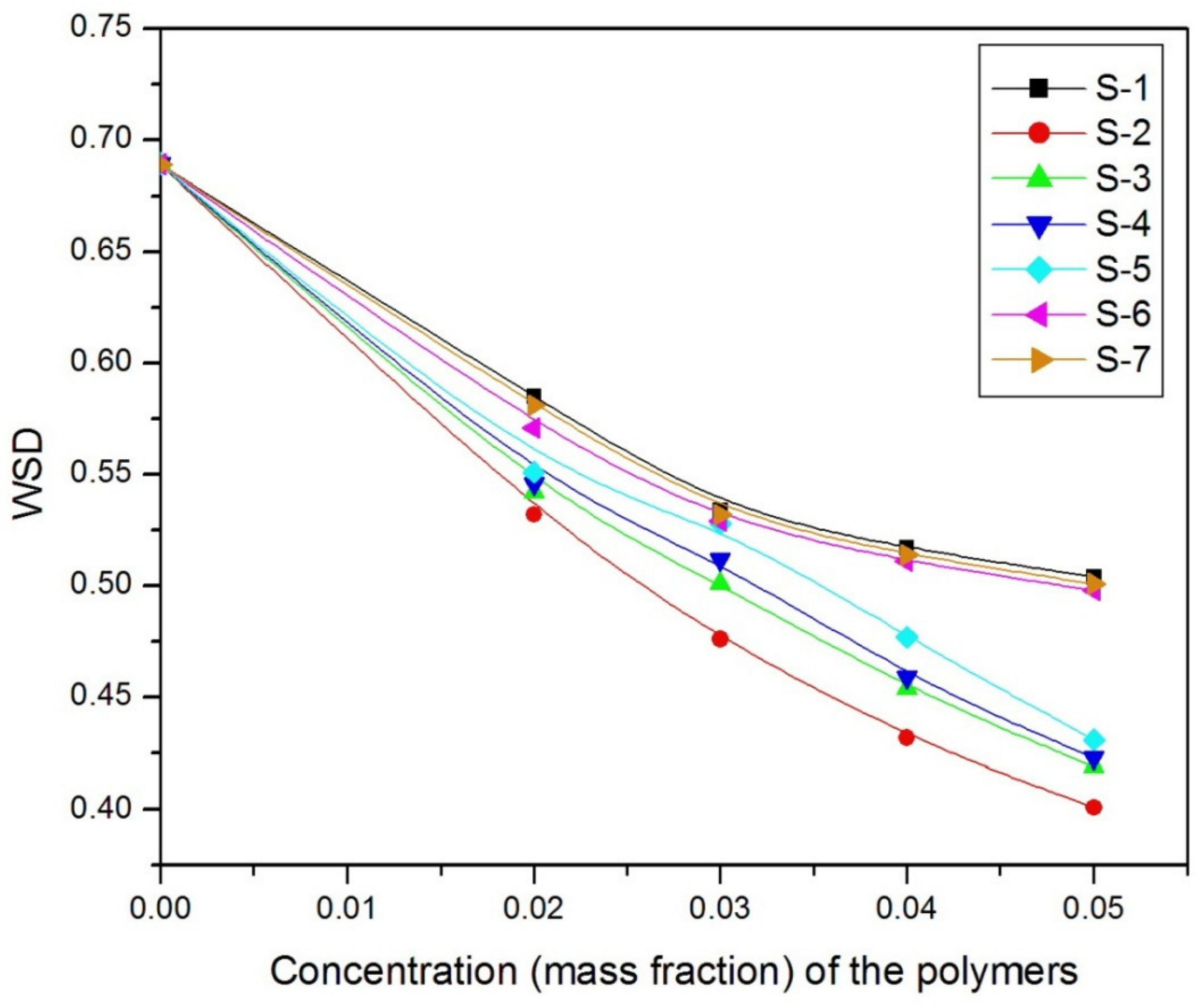
Wear scar diameter (WSD) in mm of the base oil (SN1) blended with the additives in different mass fractions (reproduced with permission from ref. [24], Copyright 2015 American Chemical Society).

**Figure 3:**
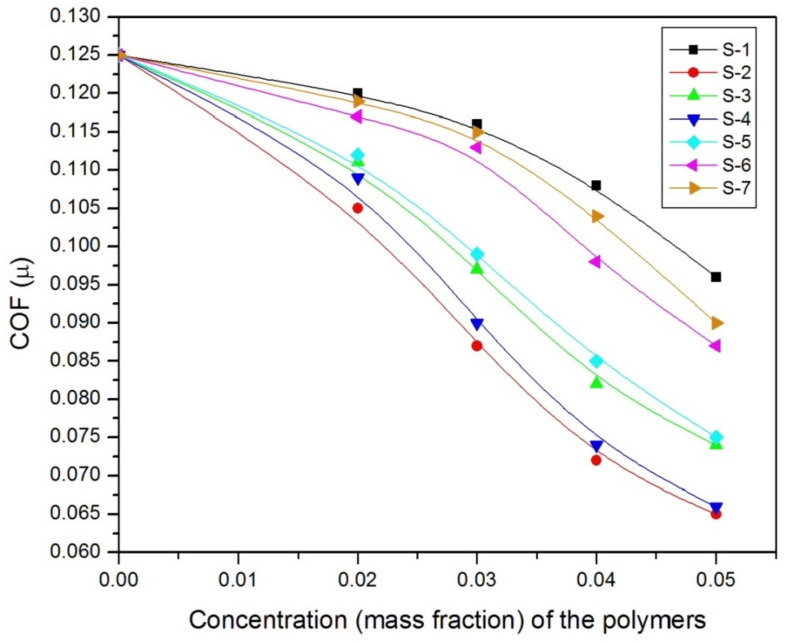
Coefficient of friction (COF) values of the SN1 base fluid blended with additives in different mass fractions (reproduced with permission from ref. [24], Copyright 2015 American Chemical Society).

The tribological properties in terms of wear scar diameter (WSD) and COF (coefficient of friction) of the lubricant compositions prepared by blending the polymers at different concentrations in SN1 oil were determined by a four-ball wear test apparatus (FBWT) at 392 N applied load. Figure 2 and Figure 3 showed the WSD and COF values of the lubricants with different percentages of additives. As indicated by the figures, the copolymers of SBO with MA and 1-decene perform better as antiwear additives. With increasing additive concentrations, both the WSD and COF decrease. S-2 showed the highest reduction of the WSD in the SN1 base oil at a 0.05 mass fraction (41.8%), whereas the homopolymer of SBO was minimal (26.8%).

**Figure 4:**
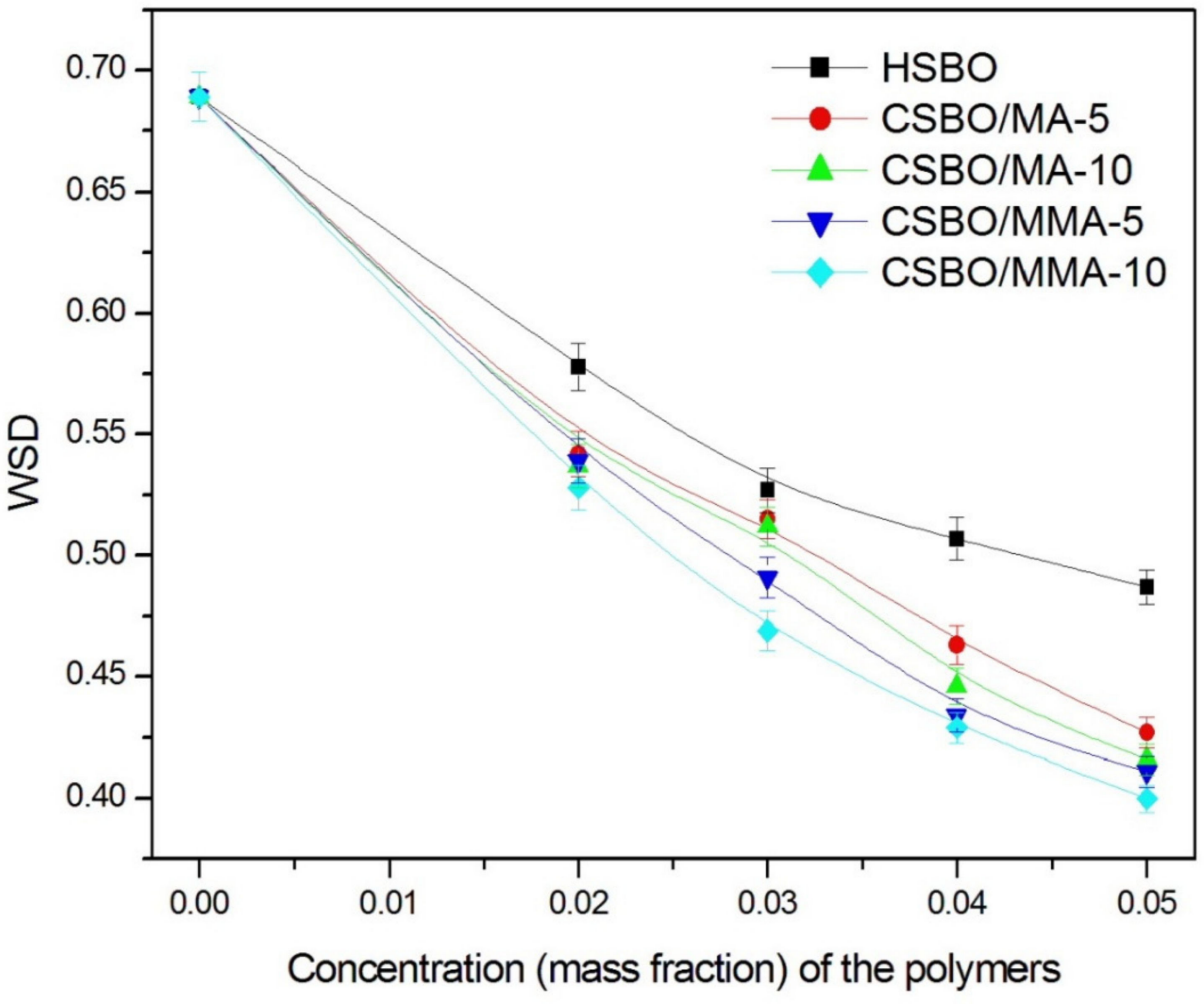
WSD (mm) values of SN70 base oil blended with additives in different mass fractions (reproduced with permission from ref. [22], Copyright 2016 American Chemical Society).

**Figure 5:**
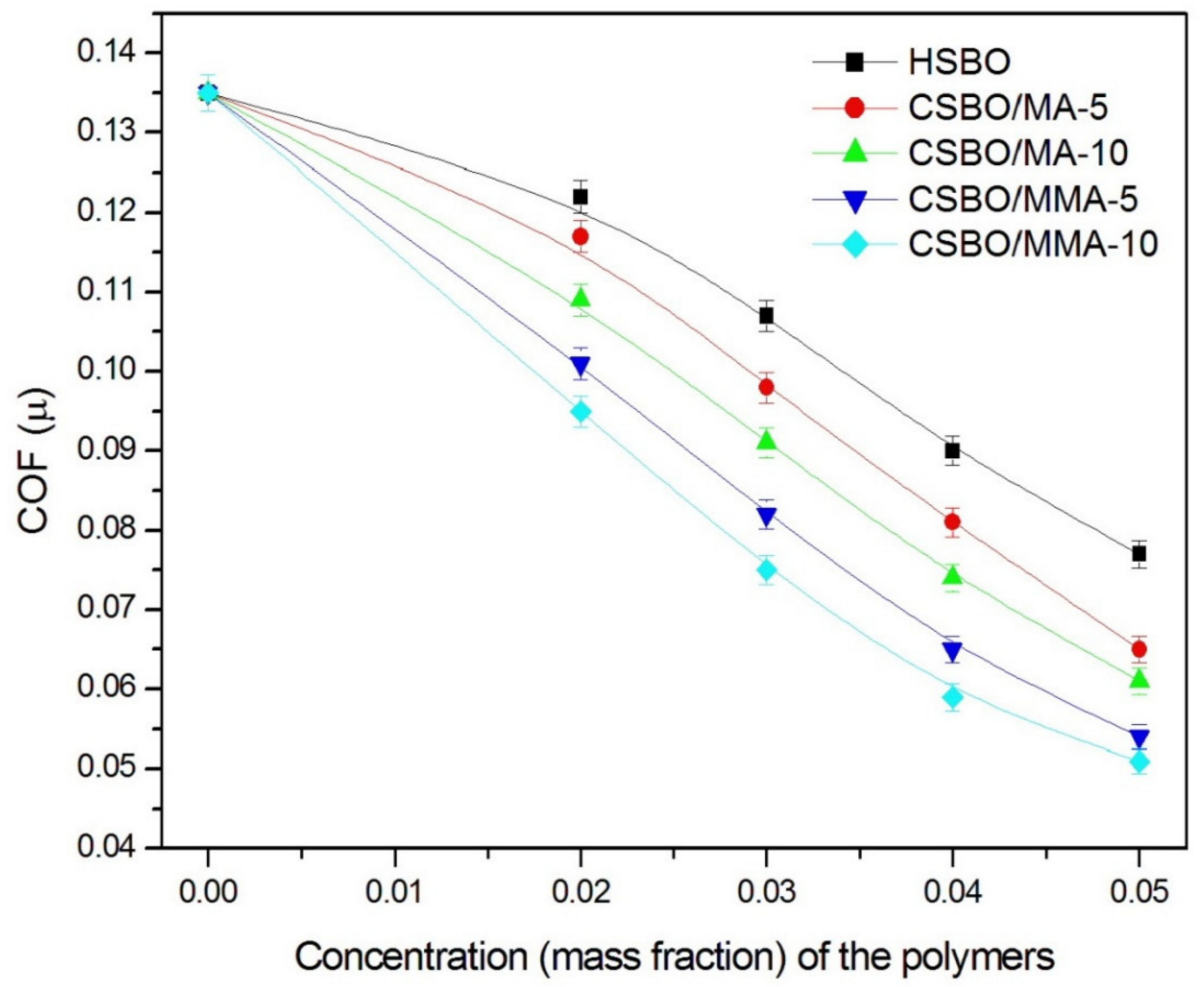
Coefficient of friction of SN70 base oil blended with the additives in different mass fractions (reproduced with permission from ref. [22], Copyright 2016 American Chemical Society).

In another study, a homopolymer of SBO and its copolymers with MA and methyl methacrylate (MMA) as a multifunctional additive for lubricating oil was synthesized by the atom transfer radical polymerization (ATRP) method [22]. Polymerization was carried out at 90 °C with microwave irradiation (MI) using anhydrous FeCl_3_ as a catalyst, diethylenetriamine (DETA) as a ligand, AIBN as the initiator, toluene as a solvent, and metallic Fe as the reducing agent. The characterization of the prepared polymers was performed by spectral (NMR, IR) and gel permeation chromatography (GPC) analysis. The evaluations of the polymers regarding their antiwear (AW) pour point depressant (PPD), and viscosity modifier/viscosity index improver (VII) performances in different mineral base oils were done by using the standard ASTM methods. The biodegradability test of the polymers was carried out by the soil burial test (SBT) method. The addition of these newly developed biodegradable additives to mineral base stocks enhances the lubricant properties significantly. The control of polymerization through ATRP and the eco-friendly microwave irradiation process produces polymers with excellent PPD, VII, and antiwear performances. Significant enhancement of the VI and PP performances was observed by incorporating acrylates (MA and MMA) in the triglyceride backbone of soybean oil, as depicted in Table 5. The antiwear performance of the lubricants was carried out by applying a weld load of 392 N (40 kg) at 75 °C for 60 min. The diameter and rotating speed of the ball were 12.7 mm and 1200 rpm, respectively. Figure 4 and Figure 5 depicted the WSD and COF values of the formulated lubricants with different percentages of additives in mineral base oil (SN70), respectively.

**Figure 6:**
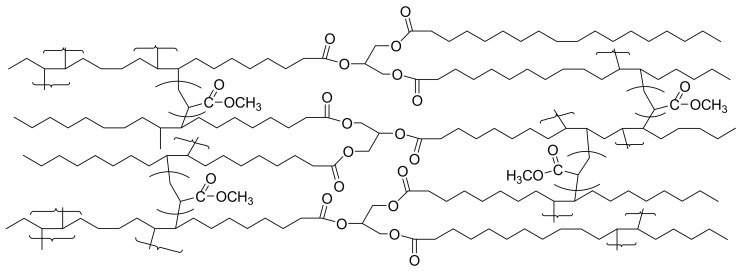
Tentative structure of copolymer of SBO and MA.

**Figure 7:**
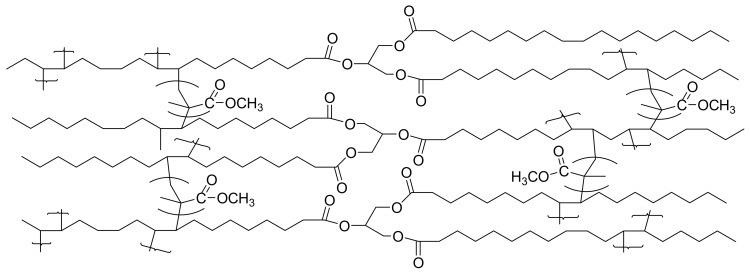
Tentative structure of copolymer of SBO and MMA.

The tentative structures of the copolymers of SBO with MA and MMA are shown in Figure 6 and Figure 7, respectively.

### 3.2. Polymers of Sunflower Oil/Derivatives

The homopolymer of sunflower oil (SFO) and its copolymerization with different mass fractions (5% and 10%) of methyl methacrylate (MMA), decyl acrylate (DA), and styrene have been carried out using the free radical technique [49]. Their characterization and performance evaluation as a pour point depressant in base oils have been investigated. The findings showed that the thermal stability of the sunflower oil-based polymers is enhanced due to the incorporation of monomers (MMA, DA, and styrene) in the backbone of the sunflower oil. The pour points of the copolymer blended base oils are better than those of the homopolymer of sunflower oil (Table 6).

**Figure 8:**
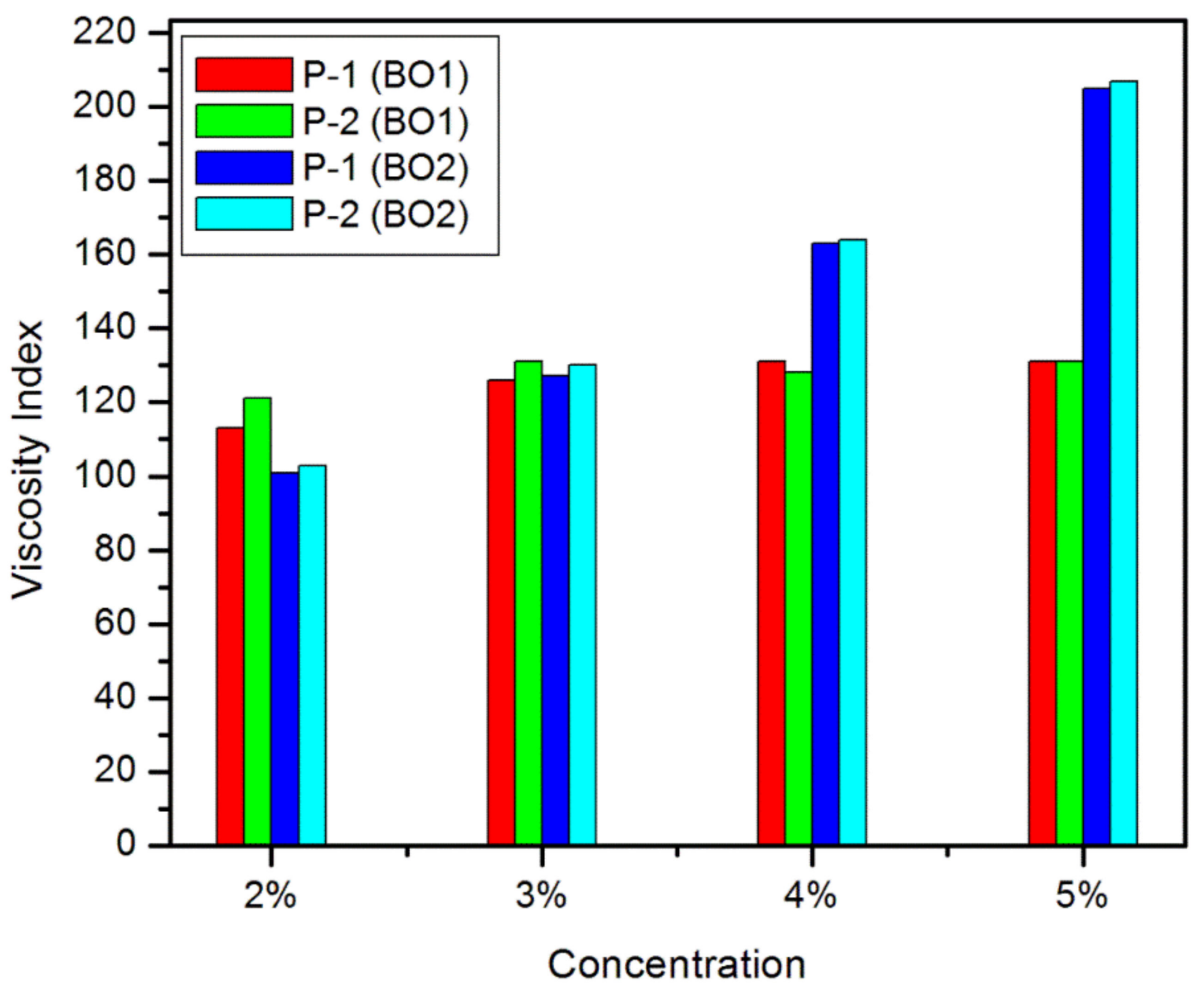
VI values of the two additive doped base oils (BO1 and BO2) at different concentrations (%*w*/*w*). [homopolymers SFO in thermal (P-1) and microwave method (P-2)] (reproduced with permission from ref. [28], Copyright 2014 Springer Nature).

**Figure 9:**
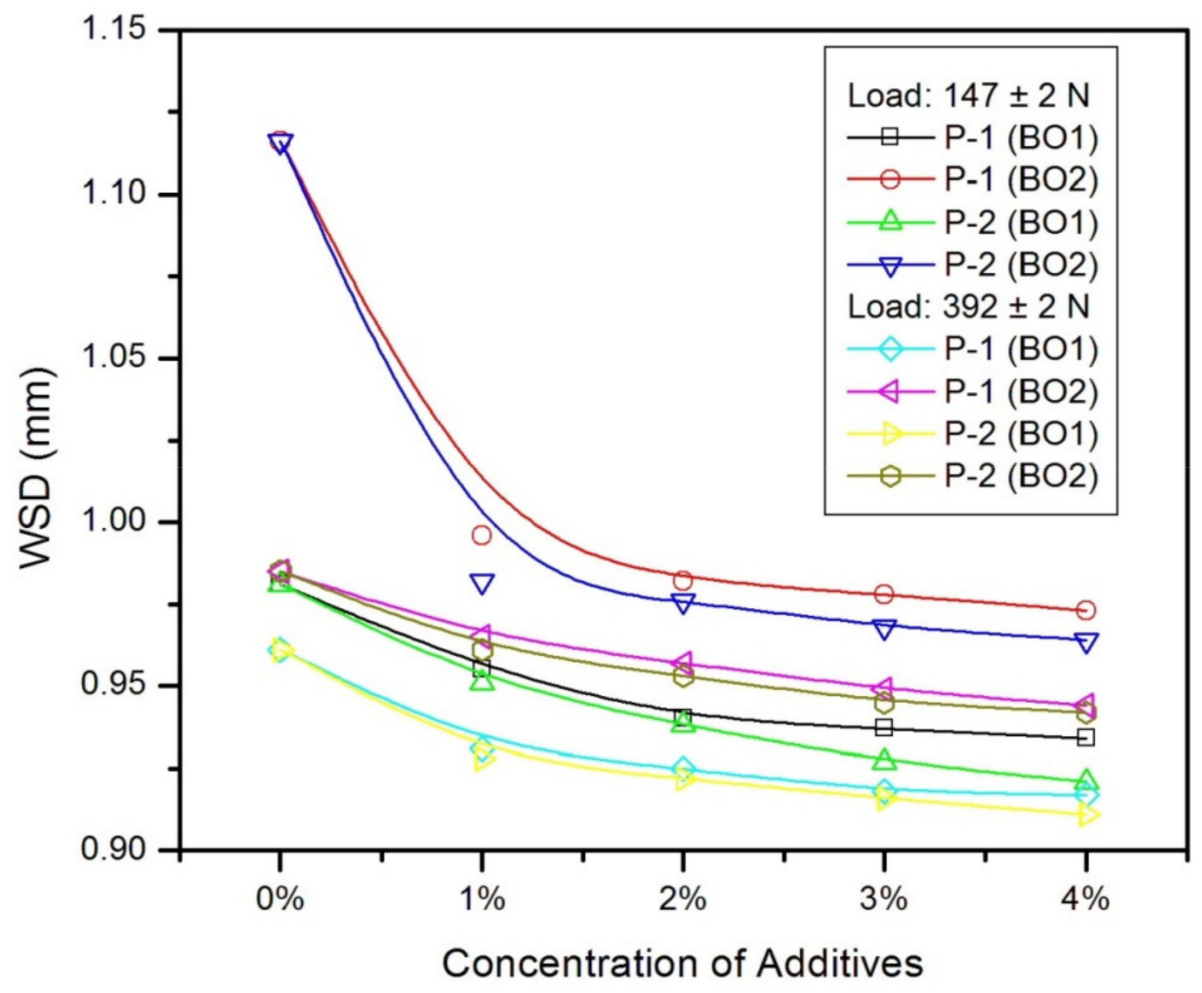
Antiwear performance of the additive doped base oils (BO1 and BO2) evaluated as wear scar diameter (mm) (reproduced with permission from ref. [28], Copyright 2014 Springer Nature).

In another study, homopolymers of SFO prepared by microwave irradiation and thermal methods using BZP as an initiator and without any solvent were used as a multifunctional additive in mineral base oil-based lubricants [28]. The homopolymer prepared by the microwave-assisted method showed better performance particularly as a viscosity index improver, pour point depressant, and antiwear additive, compared to the thermally prepared homopolymer. The viscosity index value and the WSD data of both the SFO polymer are depicted in Figure 8 and Figure 9, respectively. Moreover, the polymers showed significant biodegradability.

The copolymers of SFO with methyl methacrylate and decyl acrylate at different percentages were successfully used as a viscosity index improver in three different types of mineral base stocks [50]. In this study, a comparison of the VI performances of the SFO-based polymers with acrylate-based nonbiodegradable polymers was carried out. The results showed that biopolymeric additives have enhanced VI performances in all the base stocks compared to acrylate-based polymers in all concentrations. The VI data are given in Table 7.

**Scheme 2:**
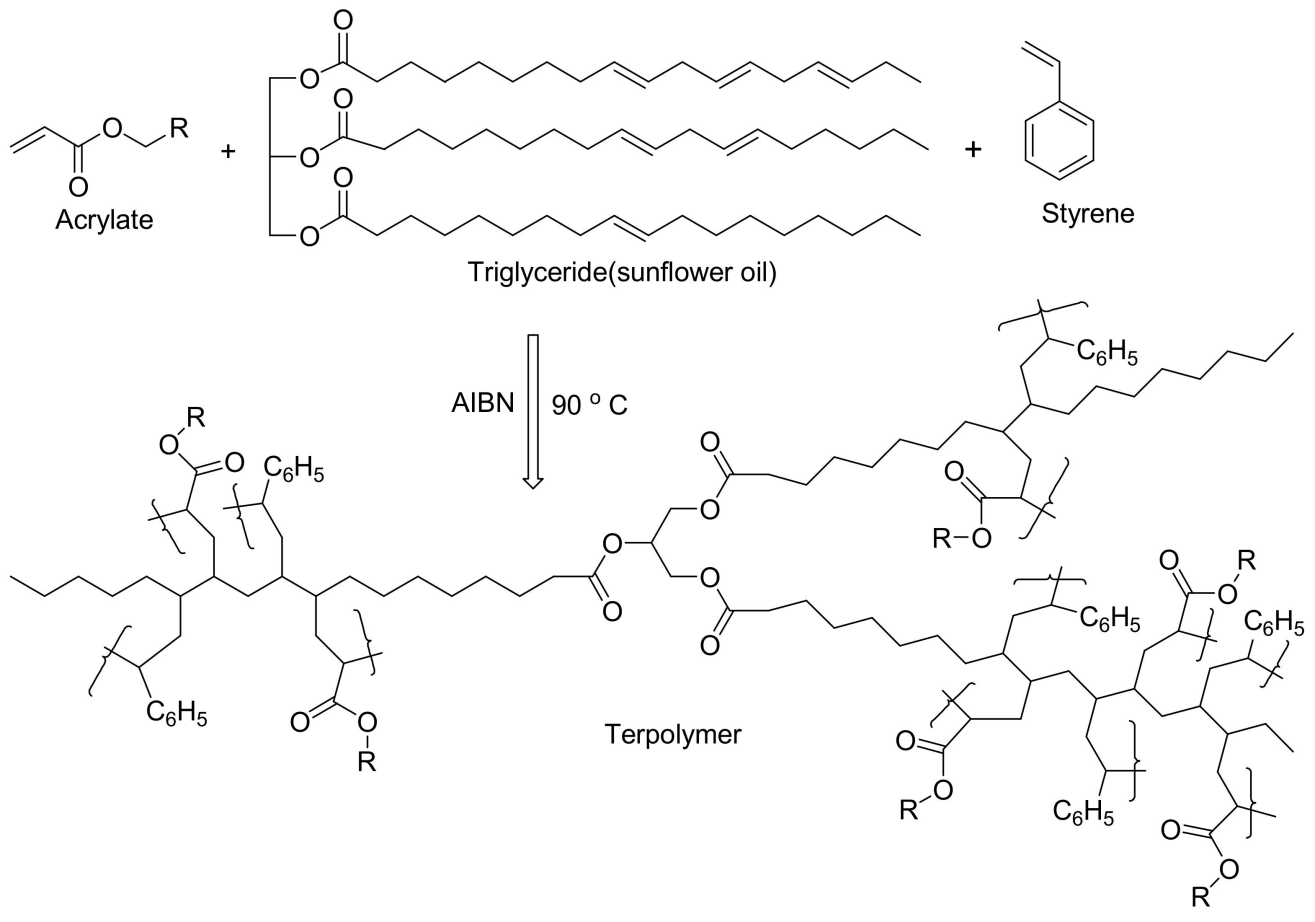
A probable structure of the terpolymer.

**Figure 10:**
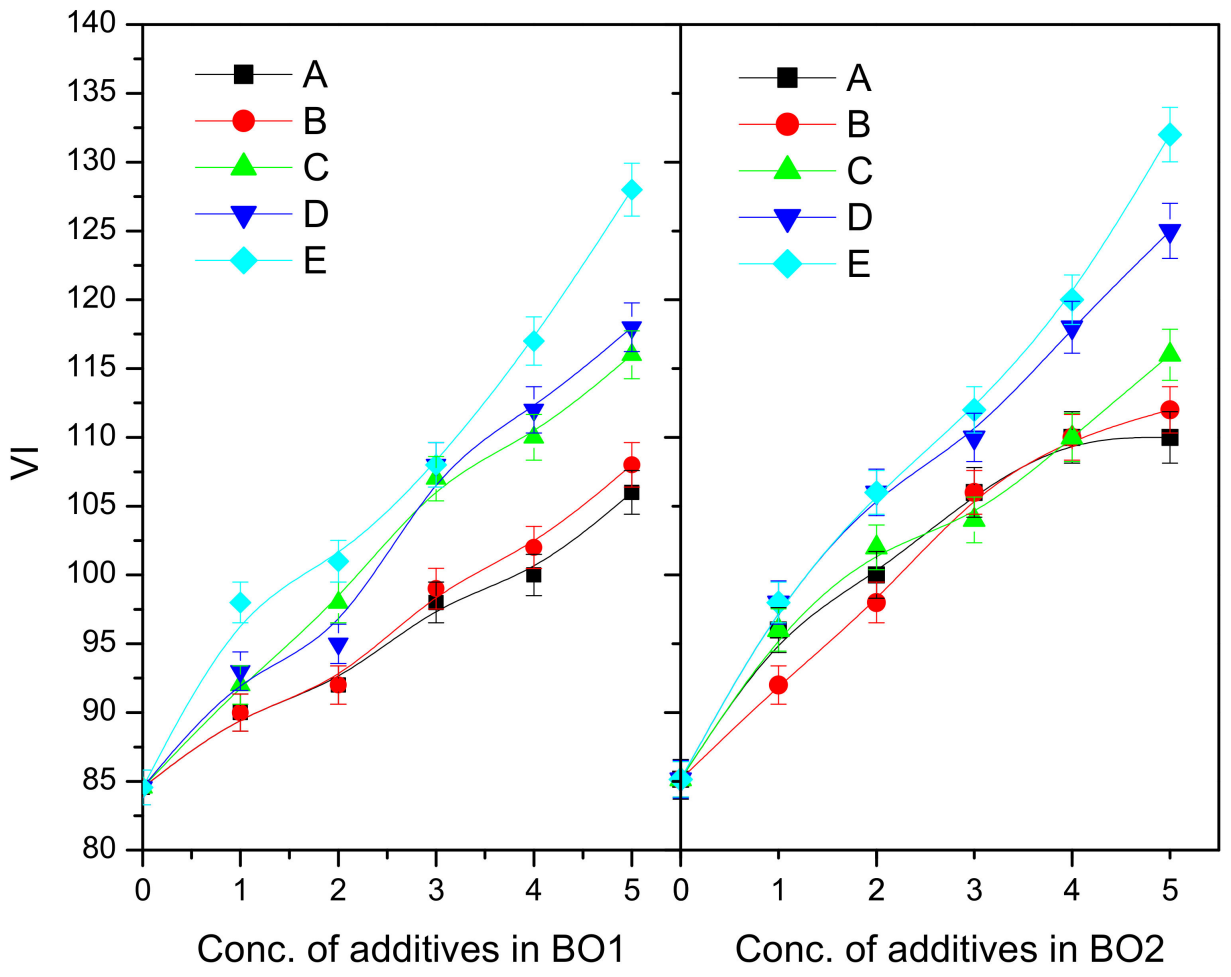
Variation of viscosity index (VI) of the base oils (BO1 and BO2) blended with the polymers at different concentration levels (% in *w*/*w*) (reproduced with permission from ref. [29], Copyright 2016 Springer Nature).

**Figure 11:**
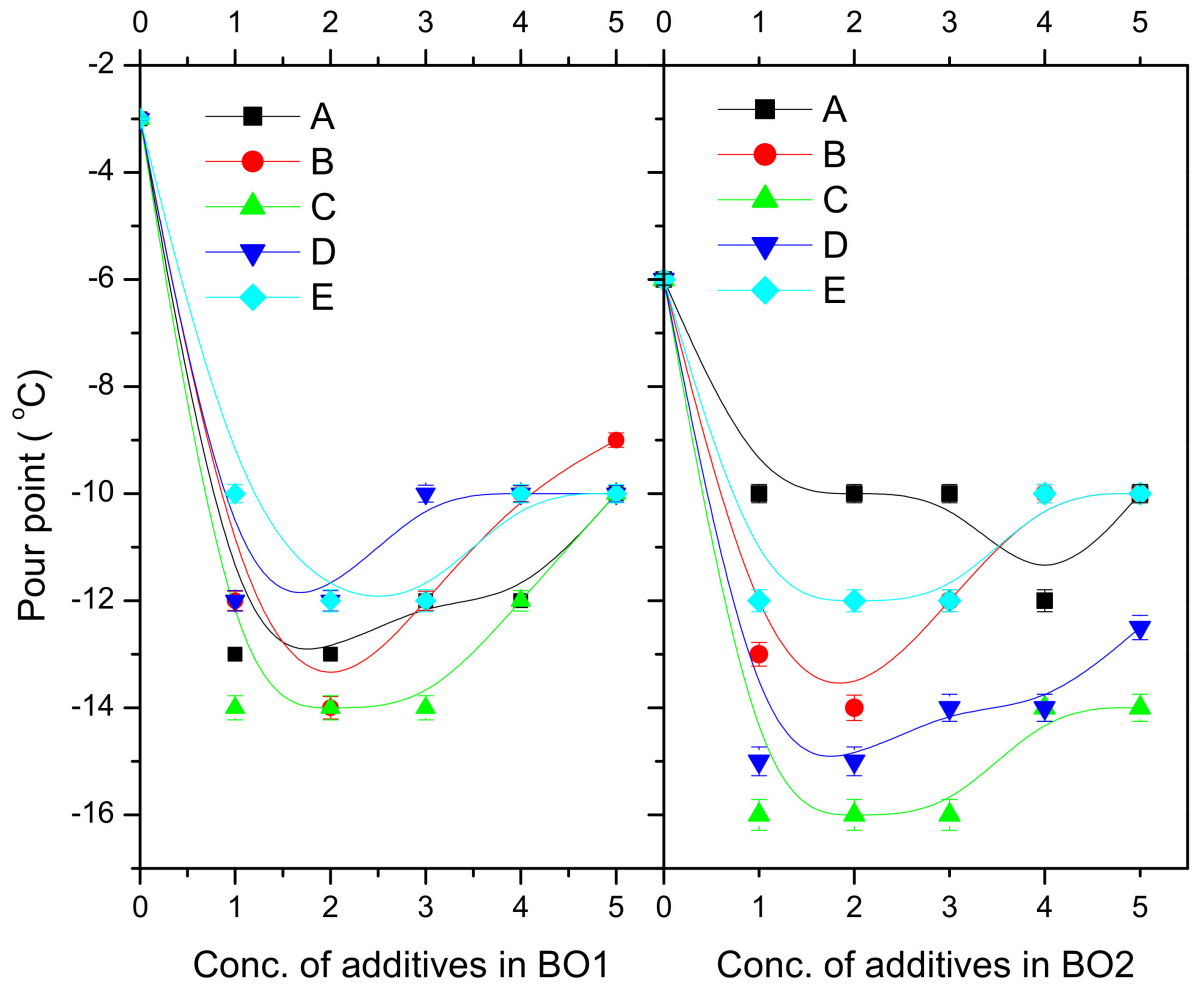
Variation of pour point of the base oils (BO1 and BO2) blended with additives at different concentration levels (% in *w*/*w*) (reproduced with permission from ref. [29], Copyright 2016 Springer Nature).

SFO-based terpolymers prepared by mixing an alkyl acrylate (octyl/decyl/dodecyl) with SFO and styrene in different ratios through free radical polymerization showed multifunctional performances like viscosity index improver (VII), pour point depressant (PPD), and antiwear when blended in different mineral base oils [29]. The image of the work is given in Scheme 2. In this work, three terpolymers have been prepared by blending octyl acrylate (OA), SFO, and styrene at different weight ratios (1:1:1,3:1:1, and 2:1:1) separately to get three polymers designated by A, B, and C, respectively. Another two polymers consisting of different alkyl acrylates [decyl acrylate (DA) and dodecyl acrylate, (DDA)], SFO, and styrene at the ratio of 2:1:1 (*w*/*w*) have also been prepared, which are designated by D and E, respectively. The investigation disclosed that an effective terpolymeric additive was formed when the ratio of acrylate, sunflower oil, and styrene was 2:1:1. The viscosity index values and pour point values are depicted in Figure 10 and Figure 11, respectively. The antiwear properties in terms of WSD were evaluated by FBWT following the ASTM D 4172-94 method. In this experiment, 392 N (40 kg) load at 75 °C for 60 min was applied. The WSD data of all the terpolymers are given in Table 8. The results indicated that when increasing the alkyl chain length, the tribological and VI performances of the prepared terpolymers increased, whereas the PPD performance decreased.

### 3.3. Polymers of Castor Oil/Derivatives

**Figure 12:**
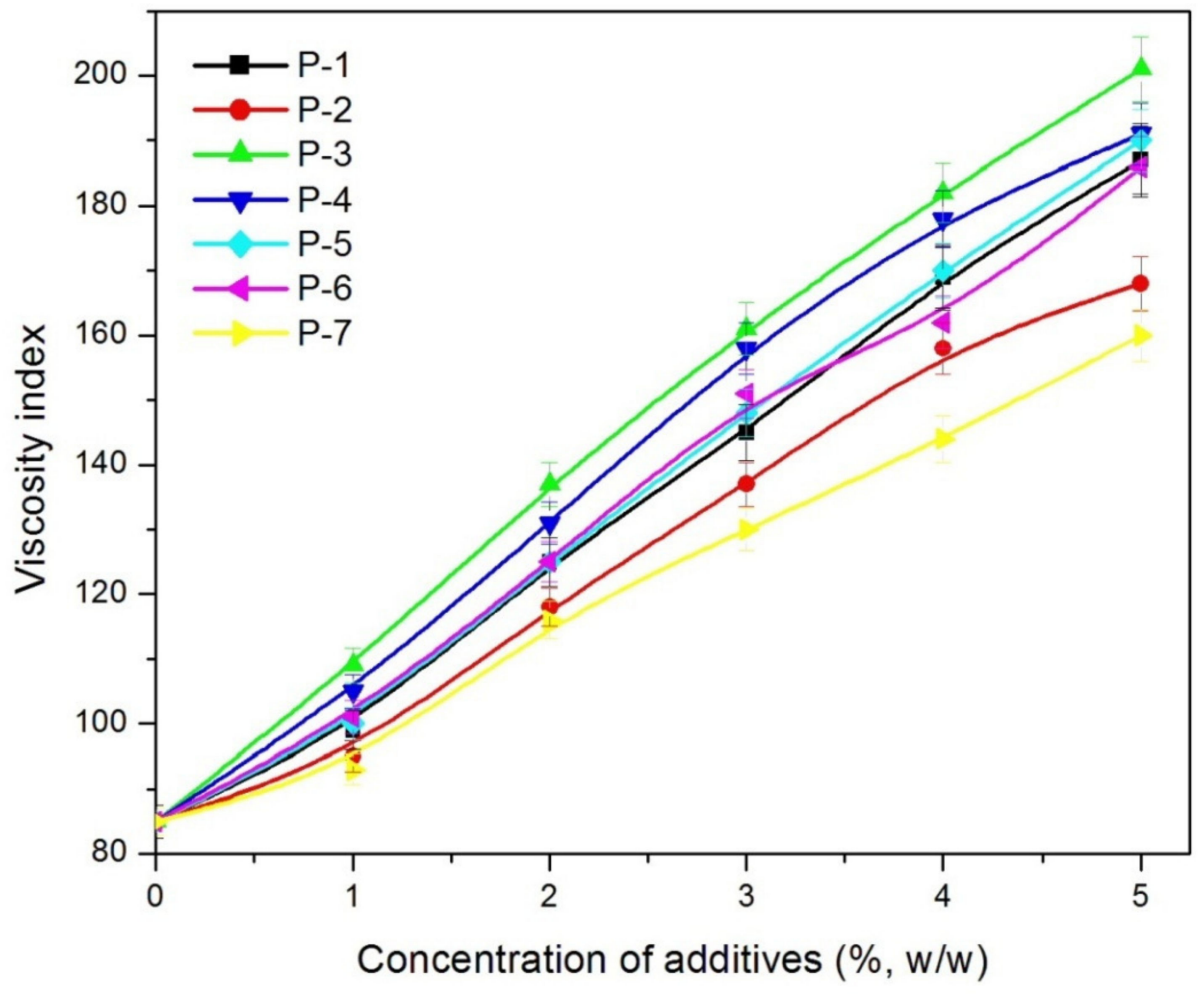
Variation of viscosity index of base oils blended with additives at different concentrations. P-1: Homopolymer of DA; P-2: Copolymer of DA + 5% CO; P-3: Copolymer of DA + 10% CO; P-4: Copolymer of DA + 15% CO; P-5: Copolymer of DA + 20% CO; P-6: Copolymer of DA + 25% CO; P-7: Homopolymer of CO (reproduced with permission from ref. [55], Copyright 2016 Springer Nature).

**Figure 13:**
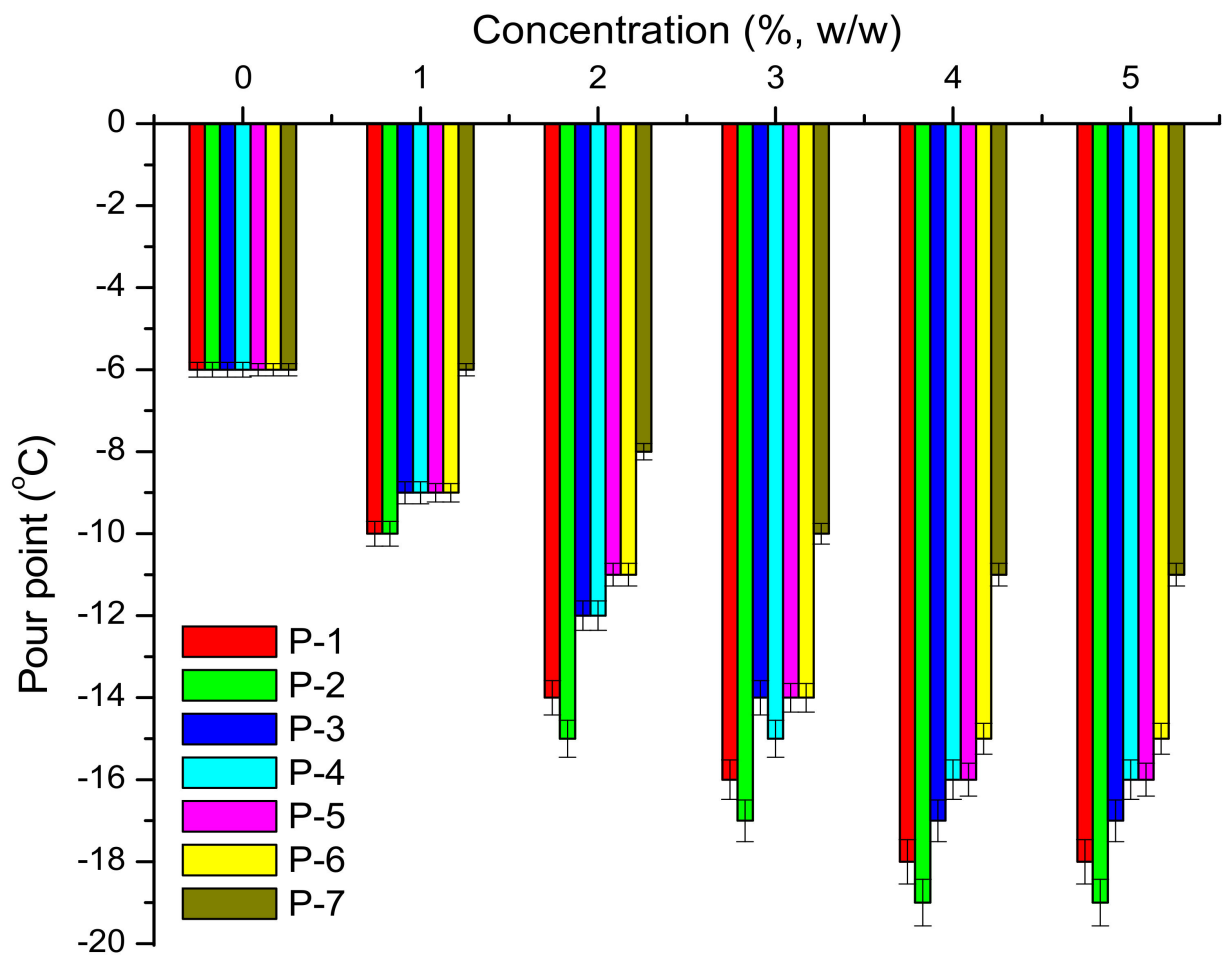
Pour points of the lubricant at different additive concentration levels (reproduced with permission from ref. [55], Copyright 2016 Springer Nature).

**Figure 14:**
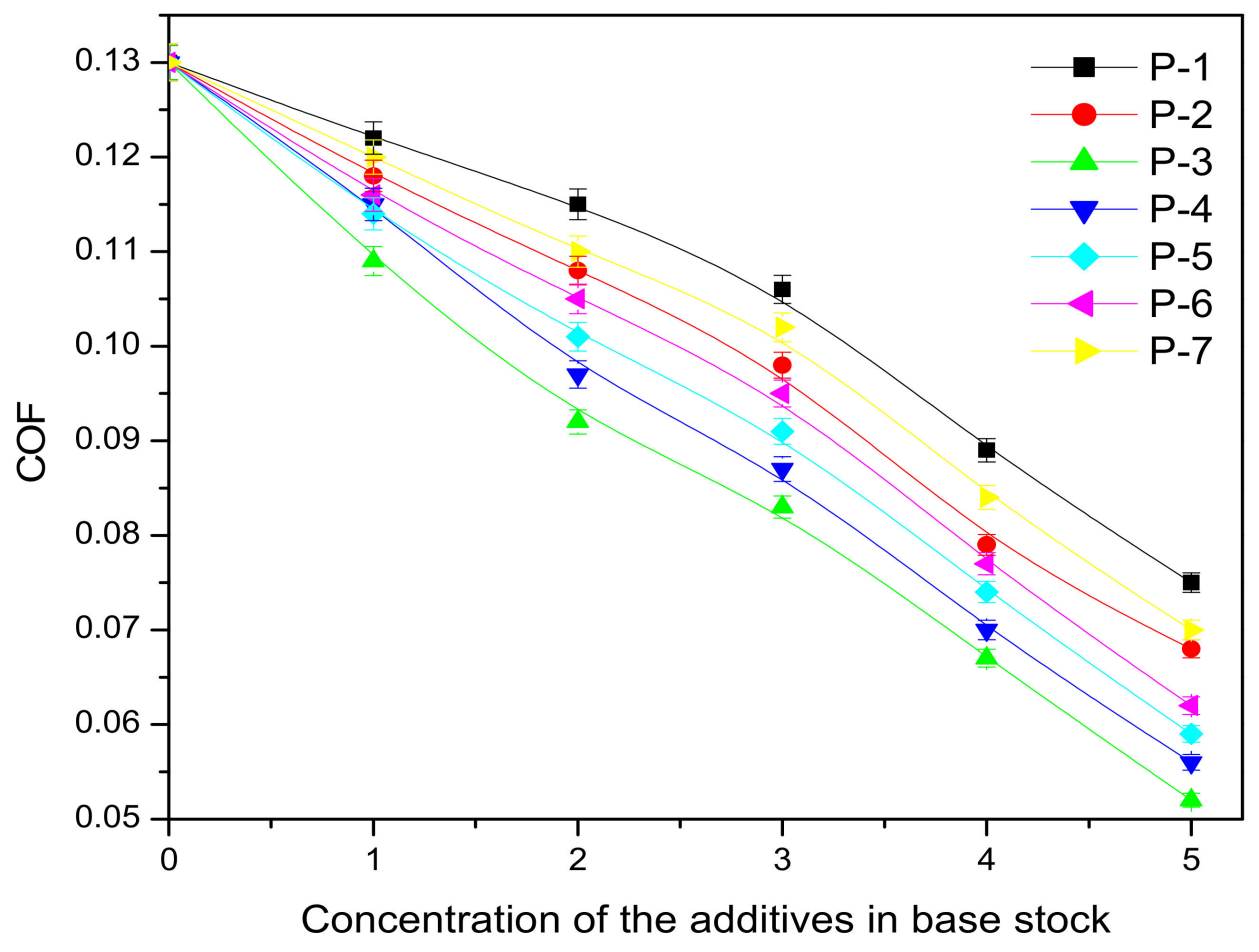
Coefficient of friction (COF) of the lube oil blended with additives at different percentages (*w*/*w*) (reproduced with permission from ref. [55], Copyright 2016 Springer Nature).

**Figure 15:**
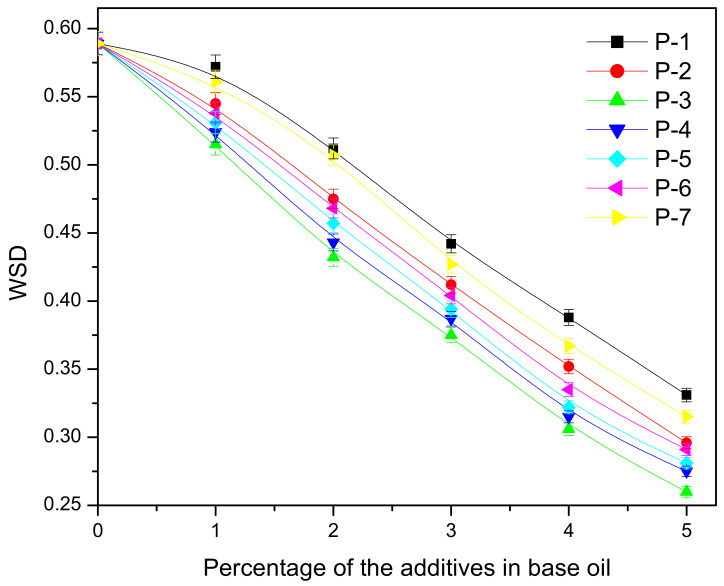
Wear scar diameter (WSD) of the lube oil blended with additives at different percentages (*w*/*w*) (reproduced with permission from ref. [55], Copyright 2016 Springer Nature).

Castor oil, which is nonedible and contains a higher percentage of triacylglycerols of ricinoleic acid (85–95%), can be used as an excellent potential feedstock to prepare a wide variety of materials such as biolubricants (base stock/additive), paints, adhesives, pharmaceuticals, cosmetics, paper, rubber, and agrochemicals [51,52,53,54]. The oil’s polar functional groups make it efficient to prepare lubricant additives, especially for antiwear and PPD performances. In our earlier studies, the homopolymer of castor oil and its copolymers with dodecyl acrylate (DDA) at different percentage levels (5, 10, 15, 20, and 25%) were synthesized by a free radical technique, and the additive performances (VII, PPD, antiwear) were evaluated by blending them with mineral base stocks [55]. The viscosity index and pour point values of polymer samples are depicted in Figure 12 and Figure 13. Tribological performance was evaluated by FBWT apparatus applying 392 N weld load. The COF and WSD data of the polymer samples are depicted in Figure 14 and Figure 15, respectively. The biodegradability test of all the polymer samples was carried out through the soil burial test (SBT). The findings showed that the pour points, viscosity index, tribological performances, and biodegradability of the lubricants were enhanced significantly when these additives were blended, and the castor oil–dodecyl acrylate copolymer exhibited better performances compared to the other polymers.

In another experiment, the additive performance of the homopolymer of castor oil (CO) and its four copolymers with methyl methacrylate (MMA), dodecyl acrylate (DDA), 1- decene (1-D), and styrene (ST) with 10% (*w*/*w*) of each have been investigated [27]. The results obtained are mentioned in Table 9. In this study PPD, VII, and tribological performances of the lubricants with these additives at different percentages have been carried out by standard ASTM methods. The SBT method was used to test the biodegradability of the polymers. The copolymers have a better performance compared to homopolymers. The castor–ST copolymer exhibited higher VI, followed by castor–MMA copolymers. The acrylate copolymers are more effective as PPDs and antiwear additives due to their polar ends.

### 3.4. Rice Bran Oil-Based Polymer

Polymers of rice bran oil (RBO) have also been used as a lubricant additive with effective performances. Copolymers of RBO with 1-decene and decyl acrylate (DA) were prepared and effectively added as a PPD and VII in lubricating oils [56]. The values of the VII and PPD are given in Table 10. In this work, rice bran oil–DA and rice bran oil-1-decene copolymers were prepared by blending different concentrations of DA and 1-decene (10%, 20%, and 30% [*w*/*w*]) in rice bran oil. RBO + DA copolymers are more efficient compared to RBO + 1-decene copolymers as a pour point depressant and a viscosity modifier. All the polymers showed significant biodegradability.

**Figure 16:**
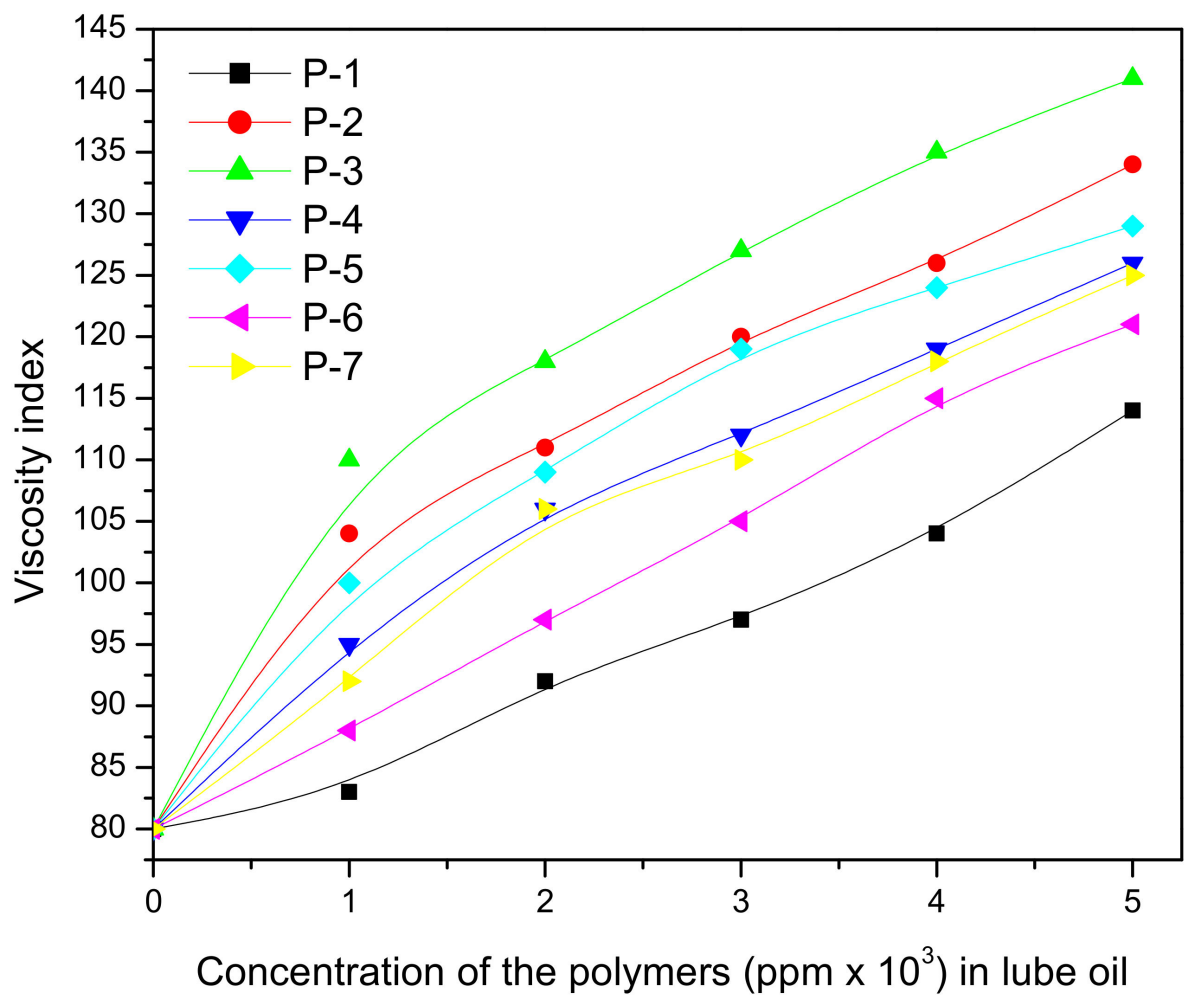
Viscosity index data of SN1 base oil blended with the polymers. P-1: homo polyisodecyl acrylate; P-2: 5% Rice bran oil + IDA; P-3: 10% Rice bran oil + IDA; P-4: 5% Peanut oil + IDA; P-5: 10% Peanut oil + IDA; P-6: 5% β -Pinene + IDA; P-7: 10% β -Pinene + IDA.

**Figure 17:**
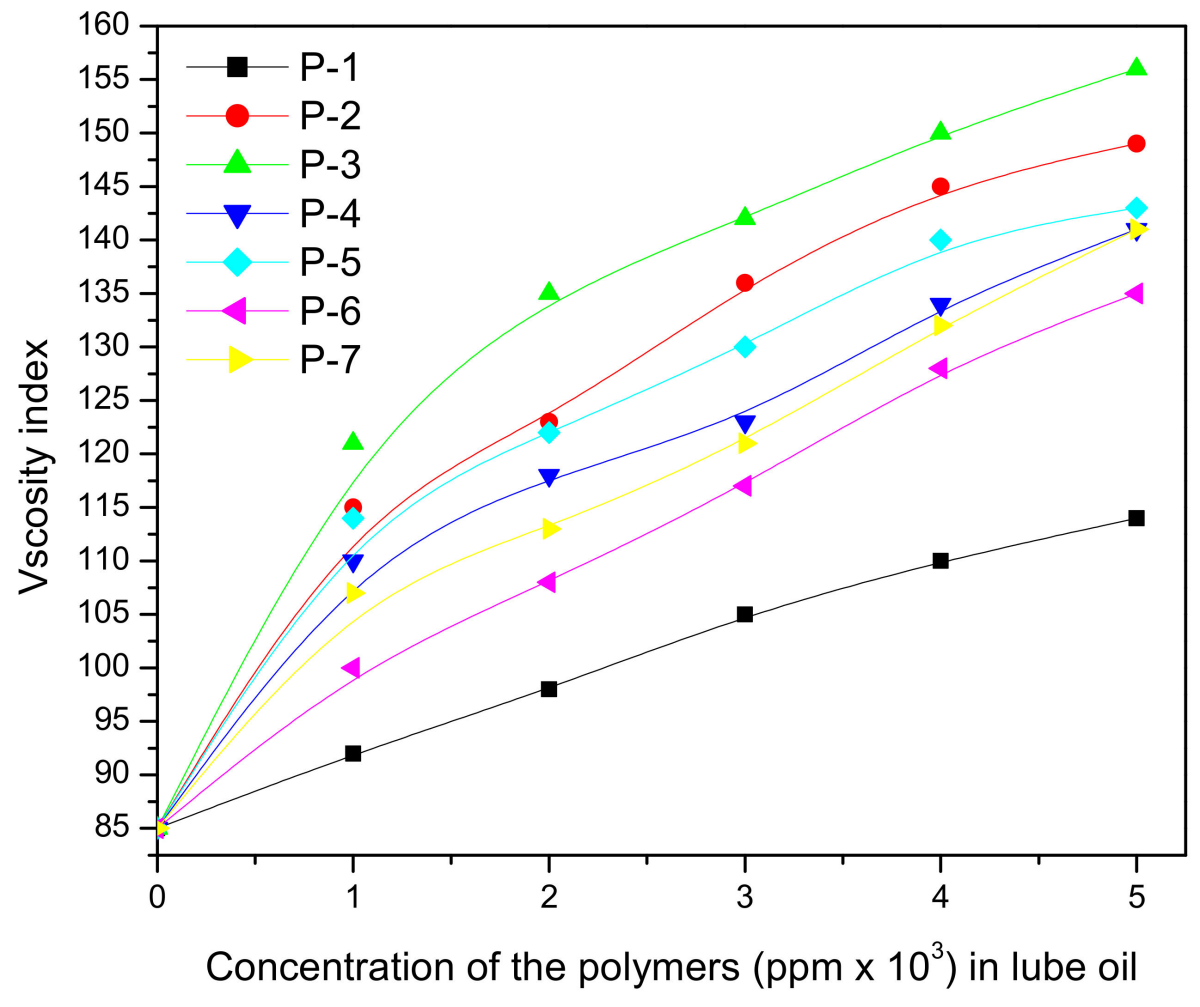
Viscosity index data of SN2 base oil blended with the polymers.

**Figure 18:**
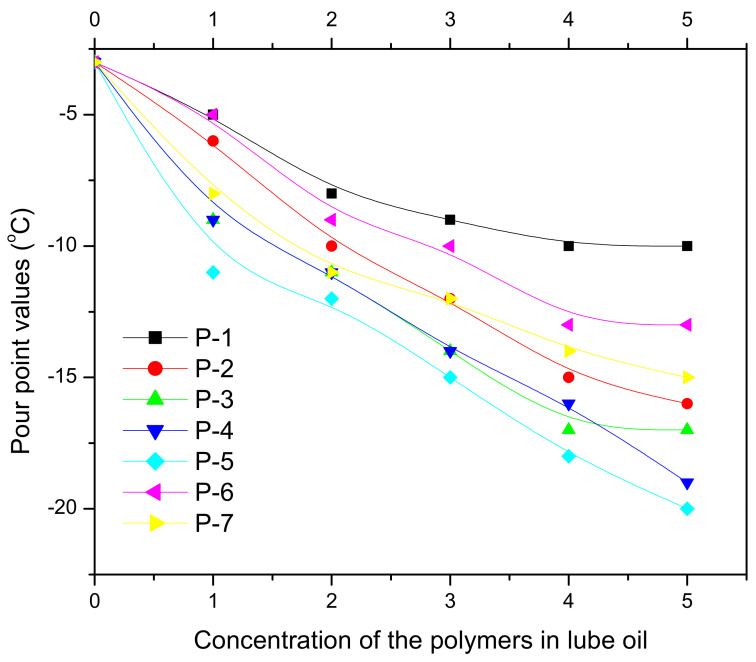
Pour point values of SN1 oil blended with the polymers.

**Figure 19:**
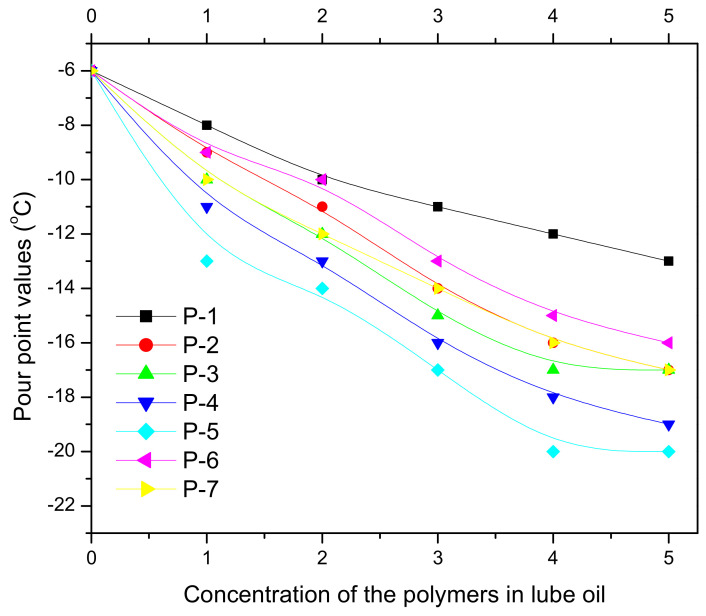
Pour point values of SN2 oil blended with the polymers.

**Figure 20:**
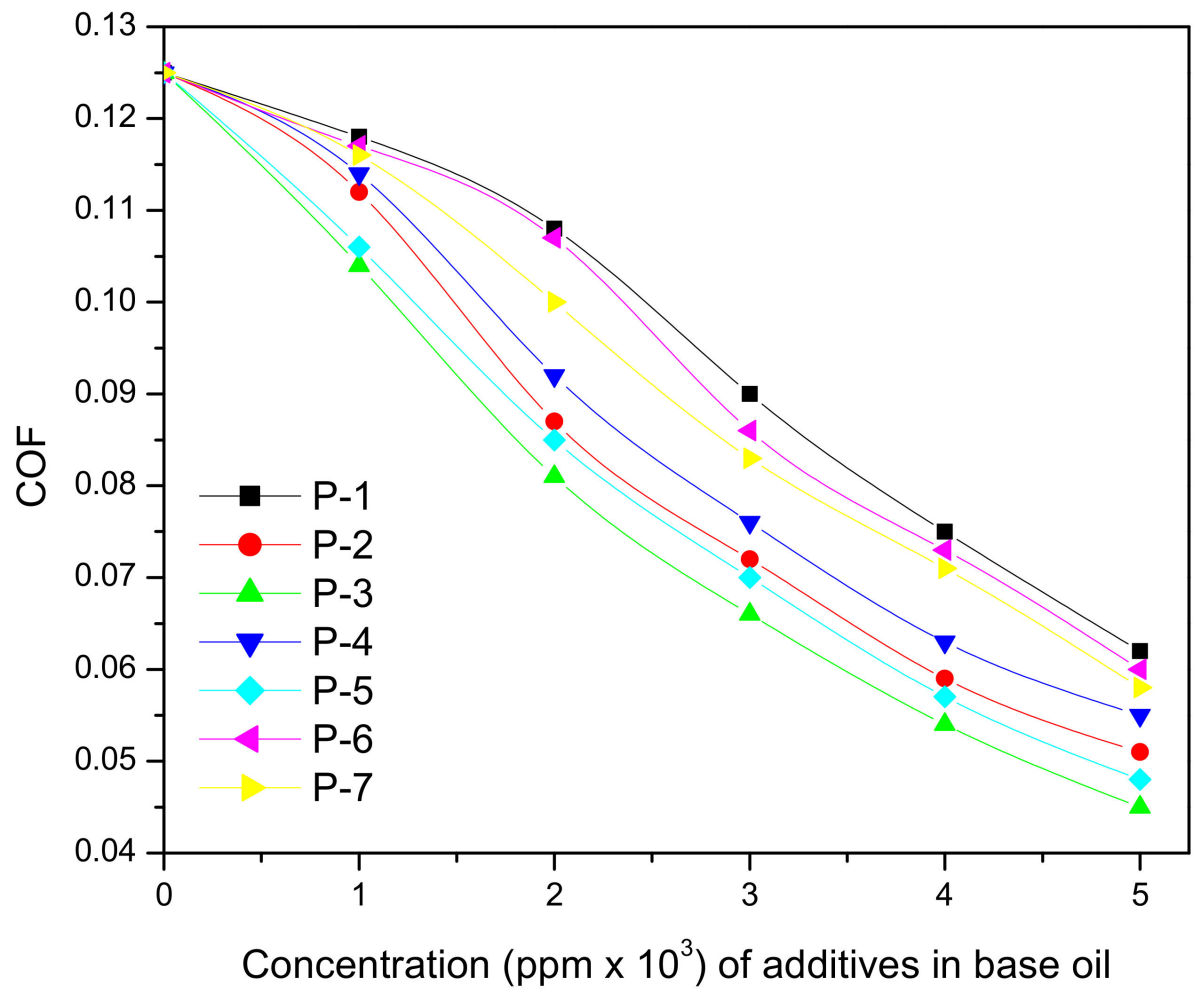
Coefficient of friction (COF) of SN1 base oil blended with the polymeric additives at different concentration levels applying 392 N weld load (reproduced from ref. [32], copyright 2017 John Wiley and Sons).

The biobased polymers synthesized by copolymerization of RBO, peanut oil (PO), and β pinene individually with isodecyl acrylate (IDA) in 5% and 10% (*w*/*w*) ratio performed as VII, PPD, and AW additives in mineral oils (SN1 and SN2) [32]. All the copolymers exhibited better PPD, VII, and tribological performances compared to the homopolymers. The viscosity index and pour point values of all the polymer samples are depicted in Figure 16, Figure 17, Figure 18 and Figure 19, respectively. The coefficient of friction (COF) data is given in Figure 20. The copolymers of RBO have higher VI values, whereas the copolymers of PO were found to be most effective as a pour point depressant. In the biodegradability test, the copolymers with higher vegetable oil/β-pinene content showed greater degradation in experimental conditions.

### 3.5. Olive Oil-Based Polymers

Olive oil is another potential candidate for preparing bioadditives. Being cheaper and having a higher percentage (about 86%) of active unsaturation, the homopolymers and copolymers of olive oil have been found to have a positive impact on lubricant performances. Copolymers of isodecyl acrylate (IDA) with olive oil, prepared by free radical pathways, were investigated as a pour point depressant (PPD) and a viscosity modifier/viscosity index improver (VII) in different base oils (mineral) by standard ASTM methods [33]. In this study, four different copolymers of olive oil and IDA were prepared with varying olive oil concentrations in the mass (2.5, 5, 7.5, and 10%). The biodegradability test (SBT method) and thermal stability (TGA analysis) of the copolymers have also been investigated. The copolymer with increased olive oil content was found to be thermally more stable and showed the best performance as PPD and VII among all the copolymers. Saha et al. evaluated the performances of lubricants additized with copolymers of olive oil and dodecyl methacrylate (DDMA) [57]. They synthesized four different copolymers of DDMA and olive oil with varying olive oil concentrations in the mass (2%, 4%, 6%, and 8%) in a focused monomode microwave oven using BZP as radical initiator. The properties, mainly viscosity indices and pour points of the lubricant compositions, have been extensively analyzed by ASTM methods. The copolymers with a higher content of olive oil indicated a higher VI and lower pour points.

### 3.6. Almond Oil-Based Polymers

In another study, the additive performances of almond oil-based polymers [58] have been disclosed. The homopolymer of almond oil and its copolymers with decyl acrylate in two different concentrations (5 and 10%, (*w*/*w*)) were prepared, and their additive performances were evaluated.

The additive performances were improved due to the incorporation of an acrylate monomer in the triglyceride backbone of almond oil.

### 3.7. Palm Oil-Based Polymers

The homopolymer of palm oil and its copolymers with decyl acrylate at different concentrations were evaluated as PPD and VII in mineral lubricants by standard ASTM methods. All the polymers showed excellent additive performances with significant biodegradability [34]. Liew et al. disclosed the application of palm oil methyl ester (POME) as a friction-reducing additive for mineral base oil [59]. Table 11 showed the wear scar diameter data of the base oil blended with POME at two different concentrations. It was further confirmed from the data that bioadditives increase the tribological performance of lubricants.

### 3.8. Jojoba Oil-Based Polymers

The research team guided by Nasser (2015) prepared a homopolymer of jojoba oil along with its six copolymers with monomers of different alkylacrylates (dodecyl-acrylate, tetradecyacrylate, and hexadecyacrylate) and different α–olefins (1-dodecene, 1-tetra-decene, and 1-hexadecene) separately with a 1:2 molar ratio [38]. The performances of these polymers as a VII and a PPD were evaluated after their characterizations. It has been found that the viscosity index increases with an increase in the alkyl chain length of both α-olefins, and acrylate monomers, while the pour point improved for additives based on alkyl acrylate.

### 3.9. Preparation of Vegetable Oil-Based Polymer Nanocomposites and Their Application as an Antifriction Additive for Biolubricants

The use of polymer nanocomposites (PNC) as an antiwear additive for lubricants has become very popular recently. With increasing environmental concerns, the use of bioresources to prepare polymer biocomposites has come into focus [60,61]. The polymerization of vegetable oils/derivatives in the presence of different organic or inorganic nanoparticles produces vegetable oil polymer- nanocomposites, which are widely applied in the automotive industry for tribological applications [62]. Bhuyana et al. [63] have disclosed the tribological properties of novel biobased nanocomposites prepared by the cationic copolymerization of conjugated low-saturated soybean oil with styrene and divinylbenzene in the presence of reactive organomodified montmorillonite (VMMT) clay of different concentrations (0%, 1%, and 5% *w*/*w*) as the reinforcing phase and modified boron trifluoride diethyl etherate (BFE) as the catalyst. The incorporation of nanoparticles in the polymer matrix improved wear-resistant characteristics and thermal stability compared to neat polymers [64]. The coefficient of friction of the composite with 1% clay concentration is 19.2% and 22.4% lower than the ones with 0% and 5% clay concentrations, respectively. Thus, the addition of clay by 1% (*w*/*w*) resulted in a nanocomposite material with superior wear behavior compared to the ones with higher and lower clay concentrations. Based on these studies, it can be said that the VMMT in the polymer matrix plays an important role in enhancing the wear behavior of the resulting nanocomposites.

## 4. Test of Biodegradability

A biodegradability test is very important for polymeric materials towards their application in diverse fields in the current global environmental consciousness. This point has to be borne in mind also for the manufacturing of additives for lubricants. Among the various methods to examine the biodegradability of samples [65], the soil burial test method and disc diffusion method using different fungal pathogens according to ISO 846:1997 standard have been applied for the above-mentioned lubricants additized with vegetable oil-based polymers [27,28,29].

### 4.1. Disc Diffusion (DD) Method

In the disc diffusion method, biodegradation was tested against different fungal pathogens, namely *Colletotrichum camelliae* (CC), *Fusarium equiseti* (FE), *Alternaria alternata* (AA), and *Colletotrichum gloeosporioides* (CG) [49]. In this method, 1.5 g of each of the polymer samples was incubated in a bacteriological incubator apparatus at 37 °C for 30 days. The glass apparatus and culture media were autoclaved before use. In preparing the culture media for fungal strains, suitable proportions of potato extract, dextrose, and agar powder were mixed and autoclaved. Fungal growth was indicated by a change in color of the culture media from yellow to black. After 30 days of incubation, polymer samples were recovered from the fungal media and washed with chloroform, purified, and dried in an open vessel. The dried samples were weighed. The biodegradability of polymer samples was also confirmed by studying the FT-IR spectral analysis and molecular weight analysis before and after the disc diffusion test of the polymer samples.

### 4.2. The Soil Burial Test (SBT)

In this method, test samples were prepared by making a film of lubricant samples with soil compost containing microorganisms and then were kept in a bacteriological incubator apparatus at pH 7.2, 38 °C, moisture 25%, and relative humidity about 60% for about 90 days. The microorganisms present in the soil help to degrade the lubricant samples. This was performed as per ISO 846:1997 standard [24]. After definite intervals, the sample was recovered, washed with chloroform, purified, and finally dried. The dried samples were weighed. The percentage weight loss (PWL) of the polymeric samples was calculated by the formula:

PWL = [(M_0_ − M_1_)/M_0_] × 100

where M_0_ is the initial mass and M_1_ is the remaining mass after SBT and subsequent drying till constant weight.

**Figure 21:**
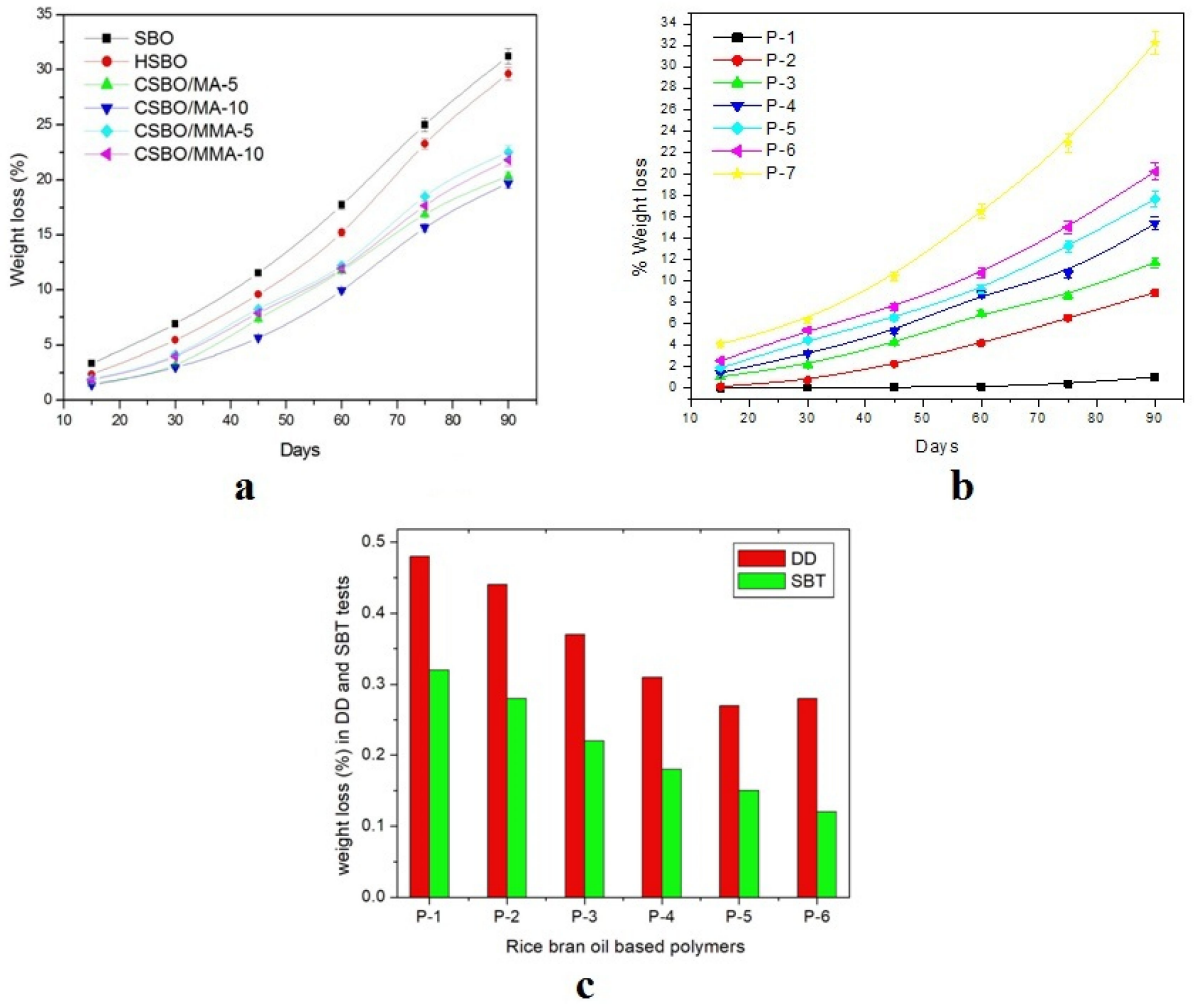
Biodegradability test results (SBT and DD); (**a**) Soybean oil-based polymers [22], (**b**) Castor oil-based polymers [55], (**c**) Rice bran oil-based polymers [56].

All the mentioned polymers showed significant biodegradability in both methods. The biodegradable nature of the lubricant samples increased with the increasing percentages of additives. The biodegradability of the homopolymers of vegetable oils showed the highest degradation. The copolymers having a higher percentage of vegetable oils showed more degradation in the tested methods. In the case of soybean oil-based polymers, polymer samples that contain a higher amount of soy oil are more biodegradable. A similar trend was observed for other kinds of vegetable oil-based polymers reported in this review. In the case of sunflower oil, the homopolymer of sunflower oil synthesized by the microwave irradiation method is more biodegradable compared to a thermally prepared one. The copolymer of rice bran oil with decyl acrylate was found to be more biodegradable compared to a rice bran oil-1-decene copolymer. Figure 21 shows the biodegradability test results of soybean oil, castor oil, and rice bran oil-based homopolymers and copolymers, as mentioned above.

## 5. Conclusions

In this short review, we highlighted some vegetable oil-based polymeric biomaterials used as lubricating oil additives. The study is significant in the present context of increasing global environmental pollution and decreasing petroleum resources. Generally, vegetable oil has poor thermal stability due to the presence of active unsaturation in the long hydrocarbon chain, which can be removed by homopolymerization or copolymerization with suitable monomers. All these polymeric materials improve the performance of lube oils. Moreover, due to their biodegradability, they are eco-friendly. Copolymers are more efficient as additives compared to homopolymers. The performances of the polymeric additives mainly depend on their morphology, which is directly linked with several factors such as type and ratio of monomers, preparation procedure, and other experimental conditions mentioned above. Copolymers of vegetable oils with alkyl acrylates (methyl acrylate, methyl methacrylate, decyl acrylate, dodecyl acrylate) showed significant enhancement in additive performances, particularly in pour point and antiwear properties compared to others. This is probably due to more polar ends in their macrostructure. On the other hand, copolymers of vegetable oils with styrene, 1-decene have comparatively higher viscosity index values. The viscosity index is directly linked with average molecular weight values which depend on the degree of polymerization of the polymers. A higher degree of polymerization and bigger hydrodynamic volumes of additive molecules in base oils enhanced VI of those copolymers to a greater extent. Another factor found that the microwave-assisted method of homopolymerization of SFO showed better performance compared to the thermal method. Therefore, a microwave-assisted method is more economical and ecofriendly for the synthesis of the homopolymer of SFO. Polymers prepared by ATRP, due to a narrow molecular weight distribution as a result of control on their polymerization, are readily soluble in base stocks and therefore more efficient as additives. The excellent additive performances of soybean oil-based homopolymers and copolymers with MA and MMA prepared by the ATRP method proved this. Nonedible castor oil is very attractive as a precursor of polymeric biomaterials because of the presence of its oxy-rich polar functional groups. The copolymer of castor oil with dodecyl acrylate, methyl methacrylate, and styrene exhibited better performances as VI, PPD, and AW additives for lube oil. Castor-styrene copolymers also exhibited a better performance as a VI than castor-methyl methacrylate copolymers. Similarly, other vegetable oils such as rice bran oil, olive oil, palm oil, almond oil-based homopolymers, and copolymers also exhibited additive performances comparable to conventional additives. Transesterified vegetable oil-based additives like palm oil methyl esters also improve tribological properties of mineral base stocks. However, being thermally more stable and of multifunctional character, the vegetable oil-based polymeric additives have some advantages. One challenge in their application is that the solubility of the additives with base stocks is not satisfactory. To improve solubility, sometimes proper diluents are added and the composition is heated to 60 °C to 70 °C with vigorous shaking. Another limitation is that the pour points of the lubricants additized with homopolymer of vegetable oils are a little bit lower compared to commercial acrylate-based additives. The pour points of lubricants blended with acrylate copolymers of vegetable oils are comparable with commercial lubricants. However, these bioadditives may be a potential green alternative to commercially available additives in the formulation of the eco-friendly lubricant composition and there is ample scope for research exploring this area.

## Figures and Tables

**Table 1:** Fatty acid compositions of commonly used vegetable oils [20].

Vegetable Oils	C12:0	C14:0	C16:0	C18:0	C16:1	C18:1	C18:2	C18:3	Others
Soybean oil	-	-	11–12	3	0.2	24	53–55	6–7	-
Sunflower oil	-	-	7	5	0.3	20–25	63–68	0.2	-
Rapeseed oil	-	-	4–5	1–2	0.21	56–64	20–26	8–10	9.1 (20:1)
Palm oil	-	1	37–41	3–6	0.4	40–45	8–10	-	-
Rice bran oil	-	-	20–22	2–3	0.19	42	31	1.1	-
Cottonseed oil	-	1	22–26	2–5	1.4	15–20	49–58	-	-
Coconut oil	44–52	13–19	8–11	1–3	–	5–8	0–1	-	-
Corn (Maize) oil	-	-	11–13	2–3	0.3	25–31	54–60	1	-
Peanut/Groundnut	-	-	10–11	2–3	0	48–50	39–40	-	-
Sesame oil	-	-	7–11	4–6	0.11	40–50	35–45	-	-
Safflower oil	-	-	5–7	1–4	0.08	13–21	73–79	-	-
Karanja oil	-	-	11–12	7–9	–	52	16–18	-	-
Jatropha oil	-	1.4	13–16	6–8	–	38–45	32–38	-	-
Rubber seed oil	-	2–3	10	9	–	25	40	16	-
Mahua oil	-	-	28	23	–	41–51	10–14	-	-
Tung oil	-	-	2.67	2.4	–	7.88	6.6	80.46 *	-
Neem oil	-	-	18	18	–	45	18–20	0.5	-
Castor oil	-	-	0.5–1	0.5–1	–	4–5	2–4	0.5–1	83–85 ^#^
Linseed oil	-	-	4–5	2–4	0–0.5	19.1	12–18	56.6	-
Olive oil	-	-	13.7	2.5	1.8	71	10	0–1.5	-

* Alpha-eleostearic acid. ^#^ Ricinoleic acid.

**Table 2:** Physical properties of vegetable oils [20].

Vegetable Oils	Iodine Value	Pour Point (°C)	Cloud Point (°C)	Kinematic Viscosity at 40 °C (mm^2^/s)	Flash Point (°C)	Density at 15 °C (g/cm^3^)
Soybean oil	138–143	−12	−4	29	254	0.914
Sunflower oil	125–140	−15	−9.5	36	274	0.916
Rapeseed oil	98–105	−15	−2	35	246	0.912
Palm oil	48–58	23.6	25.2	39.4	252	0.919
Rice bran oil	103	13	16	38.2	184	0.906
Cotton seed oil	90–119	−4.5	−0.5	34	234	0.918
Coconut oil	8–11	12.7	13.1	27	266	0.918
Peanut/Ground nut/Arachis oil	84–100	−7	4.5	40	271	0.903
Sesame oil	104–116	−11	−8	36	260	0.918
Karanja oil	81–90	−4	2	38.8	212	0.9358
Jatropha oil	82–98	−6	11	34	225	0.94
Rubber seed oil	104	18	25	33.89	228	0.928
Mahua oil	58–70	11	20	37.18	238	0.945
Neem oil	81	7	13	35.8	200	0.918
Castor oil	83–86	−21	−18	251	229	0.960
Linseed oil	168–204	−15	5	26–29	241	0.938
Safflower oil	145	−7	−2	28.3	260	0.914
Olive oil	75–94	−14	−11	39	177	0.918

**Table 3:** Characterization data of different vegetable oil-based polymers.

Polymers	FT-IR Peaks (cm^−1^)				NMR Peaks (δH, ppm)			Average Molecular Weights by GPC			Ref.
	Ester Carbonyl Group	C-O Stretching	Paraffinic C-H Stretching	C-H Bending	Ester Carbonyl Protons	Methyl Protons	Methylene Protons	Mn	Mw	PDI	
Homopolymer of SBO	1745.5	1164.9	2854.5–2923.9	724.2	4.117–4.315	0.858–1.607	2.002–2.769	46,400	48,500	1.05	[23]
Homopolymer of SFO	1732	1167.8	2853.5–2942.8	721.3	4.135–4.173	0.857–1.607	2.001–2.362	33,000	35,100	1.06	[23]
Copolymer of SBO with MA (10%)	1739–1743	1175.6	2856.5–2926	725–727	4.058–4.262	0.857–1.608	2.020–2.337	29,520	39,570	1.34	[22]
Copolymer of SBO with MMA (10%)	1735–1744	1171.5	2849–2930	725–727	4.080–4.278	0.820–1.585	1.965–2.304	34,030	48,840	1.43	[22]
Terpolymer of OA, SFO, Styrene (2:1:1)	1744	1051–1270	2857–2931	695–810	4.084–4.147	0.876–1.627	2.035–2.297	13,611	17,003	1.25	[29]
Terpolymer of DA, SFO, Styrene (2:1:1)	1744	1051–1270	2857–2931	695–810	4.084–4.147	0.876–1.627	2.035–2.297	13,623	20,522	1.51	[29]
Homopolymer of CO	1732.1	1169.3	2857–2922	722	4.118–4.328	0.861–1.611	2.033–2.343	7928	10,022	1.26	[27]
Copolymer of CO with MMA (10%)	1744.4	1169.3	2852–2926	724.4	4.900–3.719	0.941–1.260	1.784–2.083	40,879	50,979	1.25	[27]
Copolymer of CO with DDA (10%)	1742	1169.3	2847–2926	723.3	4.031–4.147	0.883–1.618	2.168–2.673	17,579	28,736	1.63	[27]

**Table 4:** Viscosity index and pour point values of lubricants with additives at different concentrations [24].

Sample	Base Oil	VI					Pour Point (−°C)				
		0%	2%	3%	4%	5%	0%	2%	3%	4%	5%
S-1	SN1	80	132	170	211	227	−3	−3	−7	−8	−12
	SN2	89	140	200	227	256	−6	−6	−8	−9	−15
S-2	SN1	80	113	127	135	138	−3	−6	−9	−12	−18
	SN2	89	113	133	153	177	−6	−8	−12	−15	−20
S-3	SN1	80	116	134	145	160	−3	−6	−8	−9	−15
	SN2	89	122	144	173	189	−6	−8	−10	−12	−15
S-4	SN1	80	156	199	232	240	−3	−3	−7	−9	−9
	SN2	89	150	212	234	262	−6	−6	−8	−9	−10
S-5	SN1	80	142	184	218	232	−3	−3	−7	−9	−9
	SN2	89	152	210	242	270	−6	−6	−6	−8	−10
S-6	SN1	80	162	201	232	242	−3	−3	−6	−9	−12
	SN2	89	166	211	244	272	−6	−6	−8	−10	−15
S-7	SN1	80	152	192	217	236	−3	−5	−7	−8	−12
	SN2	89	162	199	231	268	−6	−6	−8	−9	−15

S-1: homopolymer of SBO; S-2: SBO + 10% MA; S-3: SBO + 5% MA; S-4: SBO + 10% 1-Decene; S-5: SBO + 5% 1-Decene; S-6: SBO + 10% Styrene; S-7: SBO + 5% Styrene.

**Table 5:** Viscosity index (VI) and pour point values of the base oils blended with the additives at different concentrations [22].

Sample	Base Oil	VI					Pour Points (−°C)				
		0%	2%	3%	4%	5%	0%	2%	3%	4%	5%
S-1	SN70	80.05	102	135	189	207	−3	−6	−7	−8	−9
	SN150	89.02	115	142	198	220	−6	−6	−8	−9	−10
S-2	SN70	80.05	120	147	205	221	−3	−6	−9	−12	−18
	SN150	89.02	125	161	213	232	−6	−6	−9	−15	−18
S-3	SN70	80.05	128	169	211	229	−3	−6	−8	−12	−15
	SN150	89.02	132	174	223	239	−6	−6	−9	−12	−15
S-4	SN70	80.05	136	184	218	232	−3	−6	−6	−9	−12
	SN150	89.02	142	201	232	255	−6	−6	−6	−10	−15
S-5	SN70	80.05	140	199	232	240	−3	−6	−7	−8	−12
	SN150	89.02	146	212	234	262	−6	−6	−8	−9	−12

S-1: homopolymer of soybean oil (HSBO); S-2: soy oil + 5% MA (CSBO/MA-5); S-3: Soy oil + 10% MA (CSBO/MA-10); S-4: Soy oil + 5% MMA (CSBO/MMA-5); S-5: Soy oil + 10% MMA (CSBO/MMA-10); CSBO: Copolymer of soybean oil.

**Table 6:** Pour point values of the lubricant compositions [49].

Sample	Base Oils		Pour Points (°C) of Lubricants with Additives at Different Percentages			
			0%	2.5%	5%	10%
P-1	BO1	S1	−3	−6	−6	−6
		S2	−3	−3	−3	−3
	BO2	S1	−6	−9	−9	−9
		S2	−6	−9	−9	−9
P-2	BO1	S1	−3	−12	−12	−12
		S2	−3	−12	−15	−15
	BO2	S1	−6	−15	−15	−15
		S2	−6	−15	−15	−15
P-3	BO1	S1	−3	−12	−12	−12
		S2	−3	−15	−15	−12
	BO2	S1	−6	−15	−12	−12
		S2	−6	−15	−12	−12
P-4	BO1	S1	−3	−15	−12	−12
		S2	−3	−15	−12	−12
	BO2	S1	−6	−15	−12	−12
		S2	−6	−15	−12	−12
P-5	BO1	S1	−3	−15	−18	−15
		S2	−3	−15	−18	−21
	BO2	S1	−6	−18	−21	−21
		S2	−6	−18	−21	−24
P-6	BO1	S1	−3	−9	−9	−9
		S2	−3	−9	−9	−9
	BO2	S1	−6	−9	−12	−12
		S2	−6	−9	−12	−12
P-7	BO1	S1	−3	−9	−9	−9
		S2	−3	−9	−9	−9
	BO2	S1	−6	−9	−9	−9
		S2	−6	−9	−9	−9

P-1: homopolymer of SFO; P-2: copolymer of SFO and 5% MMA; P-3: copolymer of SFO and 10% MMA; P-4: copolymer of SFO and 5% DA; P-5: copolymer of SFO and 10% DA; P-6: copolymer of SFO and 5% styrene; P-7: copolymer of SFO and 10% styrene.

**Table 7:** Viscosity index data of lubricants with synthesized additives [50].

Sample	Base Oils	VI of Lubricants with Additives at Different Percentages				
		0%	2%	3%	4%	5%
S-1	BO1	80	88	81	100	166
	BO2	89	100	116	139	197
	BO3	96	96	108	113	148
S-2	BO1	80	91	93	93	151
	BO2	89	108	116	133	197
	BO3	96	101	111	113	145
S-3	BO1	80	93	107	121	173
	BO2	89	89	127	139	212
	BO3	96	100	113	122	152
S-4	BO1	80	144	182	203	226
	BO2	89	95	120	133	210
	BO3	96	101	109	112	148
S-5	BO1	80	109	184	230	262
	BO2	89	97	108	118	215
	BO3	96	98	114	121	137

S-1: poly(methyl methacrylate); S-2: poly(decyl acrylate); S-3: homopolymer of SFO; S-4: copolymer of SFO with 5% MMA; S-5: copolymer of SFO with 5% DA.

**Table 8:** Antiwear property in terms of wear scar diameter (WSD) values (in mm) of different lubricant compositions [29].

Polymer Code	Base Fluids	WSD of Lubricant in mm at Different Additive Concentrations (% in *w*/*w*)					
		0	1	2	3	4	5
A	BO1	0.982	0.965	0.955	0.93	0.891	0.846
	BO2	1.119	1.079	1.062	1.048	1.016	0.992
B	BO1	0.982	0.964	0.952	0.928	0.89	0.842
	BO2	1.119	1.078	1.061	1.046	1.012	0.984
C	BO1	0.982	0.951	0.944	0.919	0.875	0.833
	BO2	1.119	1.072	1.053	1.038	1.006	0.974
D	BO1	0.982	0.938	0.927	0.906	0.861	0.816
	BO2	1.119	1.061	1.048	1.03	0.991	0.962
E	BO1	0.982	0.919	0.908	0.885	0.844	0.802
	BO2	1.119	1.046	1.031	0.985	0.958	0.938

A:- O.A + SFO + styrene (1:1:1); B:- O.A + SFO + styrene (3:1:1); C:- O.A + SFO + styrene (2:1:1); D:- D.A + SFO + styrene (2:1:1); D:- D.D.A + SFO + styrene (2:1:1).

**Table 9:** Performance of lubricants at different additive concentrations [27].

Biopolymers	Base Stocks	Parameters for Evaluating Performance of Different Lubricant Compositions																
		VI						PPD (°C)						Antiwear Data [WSD (mm)]				
		0%	1%	2%	3%	4%	5%	0%	1%	2%	3%	4%	5%	0%	1%	2%	4%	5%
P-1	BO1	84.6	95	99.5	111	114	121	−3	−6	−8	−8	−7	−7	0.982	0.905	0.856	0.754	0.698
	BO2	85.2	90.5	95.8	102	114	118	−6	−9	−10	−11	−10.5	−10	1.119	0.989	0.925	0.798	0.719
P-2	BO1	84.6	98.4	106	118	125	136	−3	−11	−13	−13.5	−12	−12	0.982	0.804	0.756	0.655	0.59
	BO2	85.2	96.4	99	112	121	134	−6	−15	−16.5	−17	−17	−16	1.119	0.936	0.856	0.696	0.616
P-3	BO1	84.6	96.8	105	116	122	130	−3	−11	−12	−13	−12	−11	0.982	0.824	0.776	0.69	0.625
	BO2	85.2	95.8	100	109	116	128	−6	−14	−16	−16.5	−16	−15	1.119	0.951	0.882	0.722	0.631
P-4	BO1	84.6	96	104	110	123	128	−3	−9	−10	−10	−10	−10	0.982	0.885	0.836	0.748	0.678
	BO2	85.2	95	101	108	114	125	−6	−12	−14	−14.5	−14	−13	1.119	0.985	0.915	0.775	0.705
P-5	BO1	84.6	100	122	125	132	138	−3	−8	−8	−8	−7	−7	0.982	0.875	0.826	0.755	0.668
	BO2	85.2	99.4	108	121	128	138	−6	−10	−12	−12	−12	−11	1.119	0.978	0.902	0.736	0.692

P-1: Homo of CO; P-2: CO + 10% MMA; P-3: CO + 10% DDA; P-4: CO + 10% 1- D; P-5: CO + 10% ST.

**Table 10:** Viscosity index and pour point data of the lubricants with additives at different concentrations [56].

	Base Oil	Conc. (%)	Lubricants with Additives					
			P-1	P-2	P-3	P-4	P-5	P-6
VI	BO1 (85)	1	112	116	119	100	110	118
		2	112	118	125	104	112	122
		3	125	130	132	106	114	128
		4	125	135	138	118	126	135
		5	128	136	145	121	128	138
		6	134	136	146	125	129	137
	BO2 (80)	1	95	98	101	98	100	104
		2	97	100	110	109	104	110
		3	102	115	115	115	118	119
		4	105	115	123	118	122	125
		5	115	123	132	131	130	132
		6	116	128	138	137	129	138
PPD (°C)	BO1 (−3)	1	−9	−15	−15	−9	−9	−12
		2	−12	−18	−21	−9	−12	−12
		3	−15	−21	−24	−12	−15	−15
	BO2 (−6)	1	−12	−15	−18	−15	−21	−18
		2	−15	−18	−21	−18	−24	−24
		3	−15	−24	−27	−21	−18	−24

P-1: RBO + DA (10%); P-2: RBO + DA (20%); P-3: RBO + DA (30%); P-4: RBO + 1-decene (10%); P-5: RBO + 1-decene (20%); P-6: RBO + 1-decene (30%).

**Table 11:** Antiwear properties of mineral oil blended with POME additive at two different weld loads [59].

Lubrication Condition	Weld Load (N)	Average Wear Scar Diameter		
		300 N	600 N	800 N
Mineral oil (without palm oil methyl ester)	1200	0.28	1.97	2.6
Mineral oil (with 5 vol% palm oil methyl ester)	1450	0.29	1.79	2.3

## Data Availability

No new data were created or analyzed in this study. Data sharing is not applicable to this article.
